# At Early Rheumatoid Arthritis Stage, the Infectious Spectrum Is Driven by Non-Familial Factors and Anti-CCP Immunization

**DOI:** 10.3390/jcm13102796

**Published:** 2024-05-09

**Authors:** Marina I. Arleevskaya, Andrej A. Novikov, Anna R. Valeeva, Marina O. Korovina, Igor L. Serdiuk, Vladimir A. Popov, Caroline Carlé, Yves Renaudineau

**Affiliations:** 1 Central Research Laboratory, Kazan State Medical Academy, 420012 Kazan, Russia; anna-valeeva@mail.ru (A.R.V.); koporulina.mo@gmail.com (M.O.K.); dr.serdyuc@mail.ru (I.L.S.); 2 Institute of Fundamental Medicine and Biology, Kazan (Volga Region) Federal University, 420008 Kazan, Russia; 3 Institute of Artificial Intelligence, Innopolis University, 420500 Innopolis, Russia; a.hobukob@gmail.com; 4 Institute of Environmental Sciences, Kazan (Volga Region) Federal University, 420008 Kazan, Russia; 5 Institute of Physics, Kazan (Volga Region) Federal University, 420008 Kazan, Russia; vapopoff@yandex.ru; 6 Department of Immunology, Hôspital Purpan, INSERM U1291, CNRS U5051, Université Toulouse IIII, 31062 Toulouse, France; carle.c@chu-toulouse.fr (C.C.); renaudineau.y@chu-toulouse.fr (Y.R.)

**Keywords:** early rheumatoid arthritis, chronic tonsillitis, respiratory tract infections, HSV reactivation, anti-CCP

## Abstract

**Background/Objectives:** Patients with rheumatoid arthritis (RA) are prone to develop infections. **Methods:** Accordingly, 195 untreated early (e)RA patients and 398 healthy controls were selected from women in Tatarstan’s cohort to study infectious history in the anamnesis (four criteria) and in the previous year (16 criteria). Information about annual infections was collected face-to-face from year to year by a qualified rheumatologist/general practitioner and included the active use of information from medical records. **Results:** In the anamnesis, tuberculosis, and pneumonia, and in the previous year, respiratory tract infections, skin infections, and herpes simplex virus reactivation incidence were reported to be increased in eRA patients, as well as the event number and duration of acute and chronic tonsillitis. Moreover, more bacterial-suspected upper respiratory infections and urinary tract infections were retrieved in sporadic eRA patients as compared to familial eRA patients. An elevated immunization against CCP prevented respiratory tract infection in those with HSV exacerbation. Finally, associations were retrieved between infection (event number/delay) and RA indices: (i) chronic tonsillitis exacerbations with disease activity and health assessment (HAQ) in familial eRA; (ii) bacterial-suspected upper respiratory infections with the number of swollen and tender joints in sporadic eRA; and (iii) HSV exacerbation with inflammation in eRA patients with negative/low response against CCP. Here, we demonstrate the complex nature of the interplay of RA with specific infections. **Conclusions:** For the first time, differences in the patterns of annual trivial infections and their links with RA indices were found in cohorts of familial and sporadic cases of the disease. Additionally, for the first time, we identified a remarkable relationship between early RA and exacerbations of chronic tonsillitis, as well as tuberculosis in the patient’s history. Altogether, this study supports the existence of a complex interplay between infections and RA at onset driven by familial status and the presence of anti-CCP Ab at elevated levels.

## 1. Introduction

Rheumatoid arthritis (RA) is a multifactorial autoimmune disease that results from an inadequate immune response in genetically predisposed individuals; it is associated with a higher risk of developing latent or acute infections, as reported since the 1870s [1,2]. However, since that time, the direct role of infections in RA development and/or disease activity remains controversial. Several proposals have been formulated, and, some of them, supported, since the 2010s with the development of microbiome analysis [3]. First, the time of infection may be important, with a pathogenic infectious contribution that changes during the different stages of the disease and flares. Indeed, we have reported that trivial oral tract infections are more pronounced during the year preceding RA diagnosis and are then decreased within three years after RA onset [4], while herpes simplex virus (HSV) reactivation was associated with RA flares after RA onset [5]. Second, RA development and flares may result from cumulative infectious events rather than from specific bacterial or viral factors. Third, RA may be considered a syndrome, and signs and symptoms that define RA results may be driven by a complex interplay between infectious and non-infectious triggers [6]. In other words, “RA” may arise as a consequence of a number of different pathways that differ according to the RA phenotypes and infections [7]. Fourth, the genetic and immunological status at RA onset may be important, such an assertion has been reinforced by our report showing that the oral microbiome is tightly controlled in RA patients with elevated anti-cyclic citrullinated peptide antibodies (CCPs) [8].

It must be admitted that despite such a long awareness of RA and numerous attempts to link the provocation of RA with a specific infectious pathogen (we counted 27 microorganisms and viruses most often mentioned as etiological factors [3], the hypothesis about the infectious origin of the disease remains largely speculative. About half of the publications regarding this issue published in 2023 were reviews (Supplementary Figure S1). There is still a long way to go before applying the accumulated information to clinical practice. Meanwhile, given the intensive research into the triggering role of environmental factors in the development of RA, it is necessary to develop clear ideas about the interplay of specific trivial infections and RA, and the nature of their link.

We were inspired to analyze trivial infections in individuals at the preclinical and clinical RA stages and those with no family history of RA (controls) from our database, which contains clinical and laboratory information on these persons observed over time since 1997. Our goal was to compare the structure of the infectious syndrome and some clinical parameters of infections in the year before RA onset with those in the controls and to analyze the link of infections with RA indices. In this way, we might identify infections that have prognostic value in RA.

## 2. Material and Methods

### 2.1. Tatarstan’s Cohort

The cohorts were formed from persons in the Tatarstan database, which was created in 1997 at the Kazan State Medical Academy and is constantly updated. To date, the database contains information about more than 1600 individuals at preclinical (first-degree relatives of patients) RA stages, early and advanced stages of the disease, as well as healthy individuals with no family history of RA (control). With rare exceptions, the persons are Caucasians. A total of 195 early and untreated RA patients (eRAall) were included and fulfilled the disease ACR/EULAR 2010 eRA diagnostic criteria within less than one year from disease diagnosis [9]. In the cases included in the cohort before 2010, eRA was diagnosed by consensus by three rheumatologists, and RA was confirmed during follow-up using the ACR 1987 criteria for RA [10]. Based on heredity and familial information, 91 eRA patients were categorized as familial (fam) eRA when RA was reported in the 1st- and/or 2nd-degree relatives, 71 were categorized as sporadic (spo) eRA when an RA family history was excluded, and, in 33 cases, heredity information was incomplete as the patients grew up in broken families or were orphans. Information regarding the anti-CCP Ab status was available for 108 eRA patients at inclusion and/or during their follow-up. Among them, anti-CCP Ab was either negative (n = 4), at a low level (n = 37, 1–3 cut-off level), or at a high level (n = 67, ≥3× cut-off level). Controls were healthy volunteers without autoimmune and immunoinflammatory diseases in their family history, n = 398. The exclusion criteria in the cohort for eRA and healthy controls were the risk factors for infection, allergic disease with IgE detection, positivity for human immunodeficiency virus (HIV) in the history, active tuberculosis (TB), HCV, or HBV carrier status [11].

In the presented cohorts of RA patients and controls, women of close age predominated (Table 1). There was no reliable difference in laboratory and complex activity indices in the RA subgroups when analyzed using the Mann–Whitney and Chi2 tests (Table 1).

The study was approved by the Ethical Committee of the Kazan State Medical Academy, Kazan, Russia (Permit nr 15/1/2002). The written consent to conduct studies and to allow publication of the results was received from all the individuals involved in the study according to the legal requirements in Russia.

### 2.2. Infection Spectrum

The information regarding infectious criteria is presented in Table 2 and data were collected as previously described [12].

It is necessary to specify the following:

(1) The clinical and laboratory examination of the eRA patients and healthy controls was performed strictly in the absence of any sign of infection.

(2) The information on infections during the year preceding the onset of the disease in the eRA cohort was analyzed in therapy-naïve patients to avoid therapy effects.

### 2.3. Statistical Tests

For statistical analysis, the software Statistica v11 (TIBCO Software) was used.

Chi-square without Yates’ correction, one-tailed, and odds ratio tests were used to test infection incidence and infection clusters.

The Kruscal–Wallis Median Test was used to explore the impact of the infections in the patient’s history on the number and duration of annual infections and the interplay of infections. Results are presented as median and interquartile range (IQR).

The interplay of annual infection numbers, infection clusters, and RA indices was analyzed using Spearman’s correlation.

**Figure 1:**
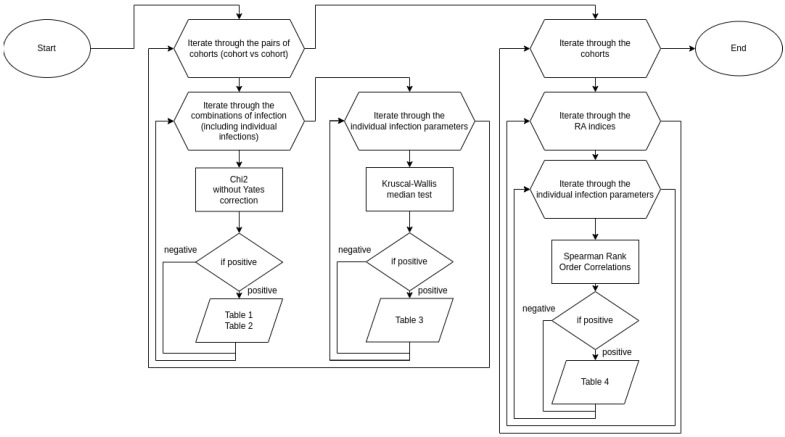
Flow chart analysis.

To control the experiment-wise error, post hoc false discovery rates and family-wise error estimations were performed. The statistical analysis algorithm is presented in the flow chart (Figure 1).

Power analysis of the chi-square test and Spearman rank order correlations results was performed using Cramer’s V effect size of independence [13,14].

Power analysis of the Kruscall–Wallis test in the aggregates of cohorts was also performed [15].

Both methods work in cases when no reliable difference was found (*p* ≥ 0.05 after post hoc analysis) or the difference was presumable (*p* ≤ 0.01 after post hoc analysis). The weak power of the obtained results 1-β < 0.5 indicates the possible detection of convincing differences in the compared parameters provided that the sample sizes are increased, which is important for these studies due to the low frequency of some infections.

For example, 1-β = 0.6, 1–0.6 = 0.4–40% probability that by increasing a cohort size the reliable difference will be obtained.

## 3. Results

### 3.1. Infectious History in Patient’s History in Early RA Patients

**Figure 2:**
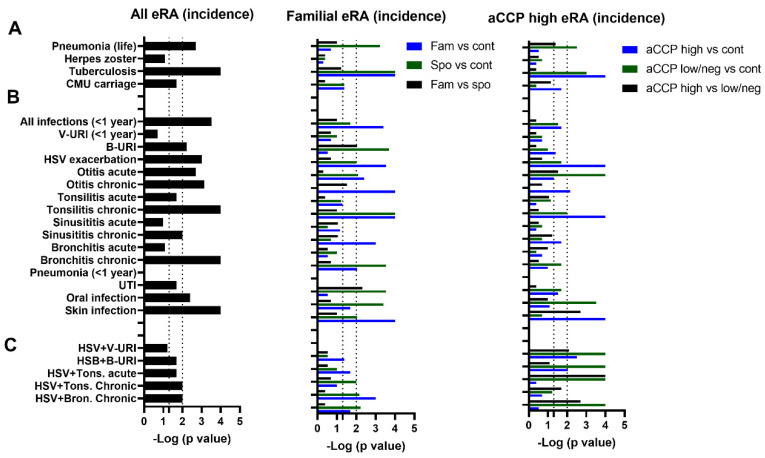
Infectious incidence in the anamnesis and in the previous year at RA onset when comparing eRA patients with healthy controls (**left**), familial from sporadic eRA patients (**middle**), and eRA with an elevated immunization status against CCP (**left**). (**A**) Infections in the anamnesis of eRA patients. (**B**) Infections reported in the previous year of eRA. (**C**) The interplay between herpes reactivation and oral tract infections. Data are presented as log10 (*p*-value) with a significant threshold fixed at *p* ≤ 0.01 corresponding to the false discovery rate post hoc. Abbreviations: eRA: early rheumatoid arthritis; Cont: healthy controls; aCCP: anti-citrullinated peptide antibodies; Fam: eRA with familial cases of RA; Spo: sporadic eRA without familial cases reported; CMU: women *Chlamydia*, *Mycoplasma*, and *Ureoplasma* species carriage; V-URI: viral-suspected upper respiratory infections; B-URI: bacterial-suspected upper respiratory infections treated with antibiotics; HSV: herpes simplex; UTI: urinary tract infections.

In order to explore whether the infectious history differs between the eRA patients and controls, 195 eRA patients and 398 healthy controls were selected. As reported in Table 1 and summarized on the left of Figure 2A, the eRA cohort was characterized, if occurring ever in life, by an increased incidence of pneumonia (*p* = 0.002) and tuberculosis (*p* = 0.0001), while, when using a post hoc test at *p* = 0.01, no differences were retrieved when considering *Herpes zoster* and, in women, *Chlamydia*, *Mycoplasma*, and *Ureoplasma* species carriage (Table 3, Figure 2).

To go further, the importance of the familial background and the anti-CCP Ab status were considered within the eRA population (Figure 2A middle and right). As compared to the healthy controls, all subgroups were at risk of tuberculosis (*p* < 0.0001), and higher pneumonia reports were retrieved in the eRA sporadic and the eRA anti-CCP neg/low subgroups (*p* = 0.006 and *p* = 0.003, respectively). Importantly, no reliable differences were observed for these parameters when eRA familial and sporadic patients were compared, nor when eRA anti-CCP high were compared to anti-CCP neg/low subgroups.

We conclude from such an analysis that a history in anamnesis of tuberculosis or pneumonia represents a susceptibility for RA development and that such effect is independent from both the RA familial/sporadic status and an elevated immunization against CCP at RA onset.

### 3.2. Infectious Incidence, Spectrum, and Associations in the Previous Year

Next (Table 4, Figure 2B left), infections reported in the year preceding the date of examination by the 195 eRA patients and the 398 healthy controls were explored revealing a global increase in the report of total infections from the eRA cohort (94.4% versus 84.7; *p* = 0.0003). When regarding in detail the infectious criteria with a threshold fixed at *p* = 0.01 after a post hoc test, differences concerned 9 out of 16 infectious criteria evaluated including bacterial-suspected URI treated with antibiotics (*p* = 0.006), herpes simplex type I/II (HSV) exacerbations (*p* = 0.001), acute and chronic otitis (*p* = 0.002 and 0.0001, respectively), chronic tonsillitis (*p* = 0.0001), chronic sinusitis (*p* = 0.01), chronic bronchitis (*p* = 0.0001), oral infection (*p* = 0.004), and skin/soft tissue infection (*p* = 0.0001).

As reported in Figure 2B middle and right, an analysis was completed by exploring the contribution of a familial RA history and an elevated immunization against CCP to the number of reports of infection in the year preceding the date of examination. As compared to controls, more infections were reported both in the familial and in the sporadic eRA sub-groups (8/16 infectious criteria in both sub-groups), as well as in the anti-CCP high eRA subgroup (4/16 infectious criteria) and the anti-CCP neg/low eRA subgroup (3/16 infectious criteria). When comparing familial/sporadic eRA patients, sporadic eRA patients have reported more bacterial-suspected URI (29.5% versus 14.3%, *p* = 0.009) and urinary tract infections (9.9% vs. 1.1%, *p* = 0.005). Regarding eRA anti-CCP related subgroups, the anti-CCP high subgroup developed more skin infections (17.9% vs. 0%, *p* = 0.002).

As part of the oral tract symptoms may be related to HSV exacerbation/reactivation, combined respiratory tract infections and HSV exacerbation were analyzed (Figure 2C, Table 1). Indeed, HSV exacerbation coupled with chronic tonsillitis (*p* = 0.01) and chronic bronchitis (*p* = 0.01) were determined to be increased in eRA patients as compared to controls. Moreover, eRA patients with HSV exacerbation and anti-CCP at elevated levels showed protection from virus-suspected URI (*p* = 0.008), acute tonsillitis (*p* = 0.0001), and chronic bronchitis infections (*p* = 0.002).

Accordingly, we conclude that eRA patients are more prone to report infectious events in the year preceding eRA onset as compared to control patients, with differences reported according to the familial/sporadic and anti-CCP status. In addition, analysis of oral tract infections combined with HSV exacerbation further retrieved that an elevated immunization against CCP is protective for this association.

Analysis of the interplay of certain infections revealed some remarkable patterns in the eRAall cohort. TB in a patient’s anamnesis was presumably linked with the more prolonged episodes of chronic tonsillitis exacerbations in the year preceding RA onset (Chi-square = 4.27, df = 1, *p* = 0.03).

Pneumonia in a patient’s anamnesis was presumably linked with a larger number (Chi-square = 3.73, df = 1, *p* = 0.05) and duration (Chi-square = 3.94, df = 1, *p* = 0.05) of chronic bronchitis exacerbations in the year preceding RA onset, which might be due to sharing of predisposing factors, not necessarily related to the characteristics of the immune response. In this cohort, the Spearman rank order correlation analysis revealed the following presumable links: the annual number of HSV exacerbations was in moderate direct correlation with the number of episodes (R = 0.5, *p* = 0.05) of chronic tonsillitis exacerbations and their duration/year (R = 0.6, *p* = 0.03); and duration/year of HSV exacerbations was in moderate direct correlation with the duration/year of chronic tonsillitis exacerbations (R = 0.7, *p* = 0.008).

### 3.3. Infectious Number, Duration, Spectrum, and Associations in the Previous Year

**Figure 3:**
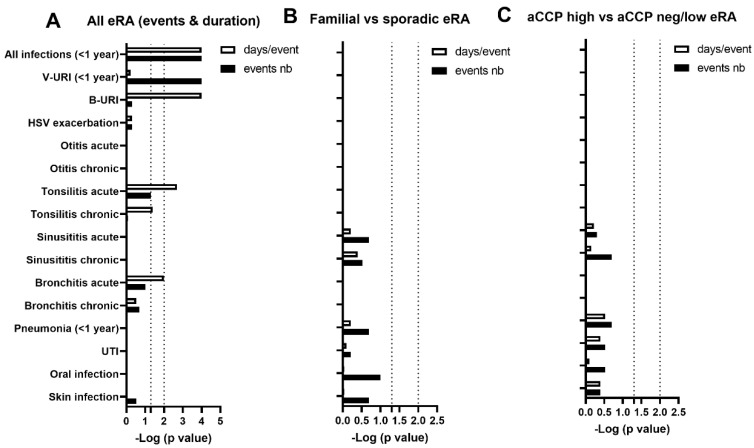
**Number of infectious events and duration per event in the previous year at RA onset.** (**A**) Comparison between eRA patients with healthy controls. (**B**) Comparison between familial and sporadic eRA patients. (**C**) Comparison between eRA patients having or not having an elevated immunization status against CCP at onset. Data are presented as log10 (*p*-value) with a significant threshold fixed at *p* ≤ 0.01 corresponding to the false discovery rate post hoc. Abbreviations: eRA: early rheumatoid arthritis; aCCP: anti-citrullinated peptide antibodies; Fam: eRA with familial cases of RA; Spo: sporadic eRA without familial cases reported; V-URI: viral-suspected upper respiratory infections; B-URI: bacterial-suspected upper respiratory infections treated with antibiotics; HSV: herpes simplex; UTI: urinary tract infections.

Next, to further test whether eRA sensitivity to infections in the year preceding the date of examination was not only qualitative but also quantitative, which may indicate an altered immune anti-infectious response, the analysis was repeated considering both the number of events and the episode duration per event (Table 5, Figure 3A). Indeed, when considering total individual infections, both parameters were increased in the year preceding the date of examination in the eRA subgroup as compared to the controls. Such an effect was related to the reporting of an increased number of events regarding viral-suspected URI (*p* < 0.0001). Moreover, episode duration was increased in eRA patients for bacterial-related URI (*p* = 0.0001), acute tonsillitis (*p* = 0.002), and acute bronchitis (*p* = 0.04). Of important note, such effects appeared to be independent of the familial/sporadic and ACPA status of the eRA subgroups.

Power analysis showed that, with increasing sample sizes, spotting significant differences where they were absent according to the Kruskal–Wallis results is mostly unlikely.

### 3.4. Interplay of Infections and RA Indices in eRA

**Figure 4:**
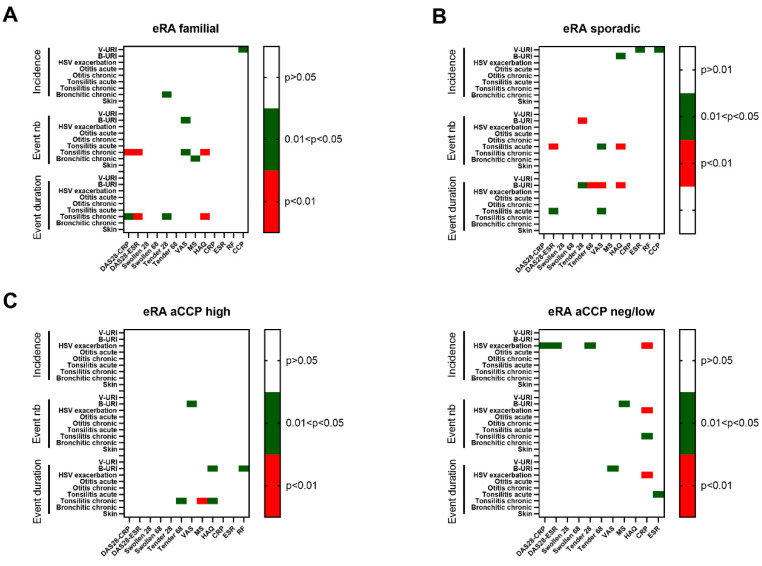
Heat map of Spearman correlation between infectious events and clinical features of RA in four subgroups at early (e)RA. (**A**) Familial eRA; (**B**) sporadic eRA; (**C**) eRA patients having an elevated immunization status against CCP; D: eRA patients with a negative or low immunization against CCP. *p*-values are indicated and considered significant when *p* < 0.01.

Next, the interplay between the previous year’s infections and eRA disease activity according to the familial/sporadic and anti-CCP status was further explored by using the Spearman rank order correlation method for quantitative values and Chi-square for nominal values with a significant *p*-value fixed at 0.01 (Table 6, Figure 4). The main results from such an analysis revealed an association between the following: (i) a remarkable correlation of chronic tonsillitis exacerbation parameters with RA indices in familial eRA, namely, the annual number of chronic tonsillitis exacerbations with DAS28-ESR (r = −0.8, *p* = 0.003) and with DAS28-CRP (r = −0.7, *p* = 0.007); the annual number of all infectious episodes and duration of all exacerbations and health assessment questionnaire values (HAQ; both correlations—r = −0.7, *p* = 0.009; r = −0.7 *p* = 0.009); (ii) bacterial-suspected upper respiratory infection annual duration with the number of tender joints (68 joint count, r = 0.8, *p* = 0.004) in sporadic eRA; (iii) the link of HSV exacerbation parameters with RA indices in eRA patients with a negative/low response against CCP and the lack of them in the eRA aCCP high group, namely, one infectious episode duration with DAS-ESR (r = 0.7, *p* = 0.1) and with a raised CRP serum level (r = 0.81, *p* = 0.0008); the CRP level also correlated with the annual HSV exacerbation numbers (R = 0.77, *p* = 0.002) and all episodes’ durations (r = 0.8, *p* = 0.0008); and (iv) in the anamnesis.

It turned out to be unexpected for us that infections in the patient’s history before RA onset appeared to impact RA indices (Figure 4). The Kruscal–Wallis median test revealed that in the eRAfam subgroup TB infection in earlier life was associated with a lower number of swollen joints (68 joint count, Chi-square = 3.93, df = 1, *p* = 0.05) and lower serum RF levels (Chi-square = 4.73, df = 1, *p* = 0.03). A similar trace was left by pneumonia in the patient’s history—eRAfam patients with this infection in the past demonstrated a lower ESR (Chi-square = 3.81, df = 1, *p* = 0.05).

**Figure 5:**
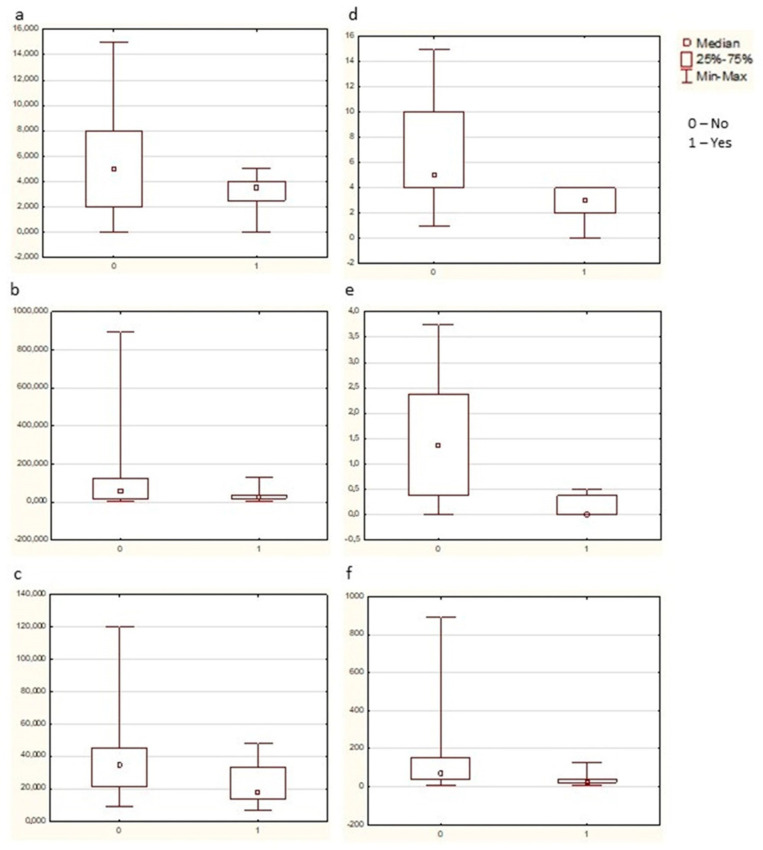
The link of RA indices and infections in eRAfam and eRAaCCP subgroups (Kruscal–Wallis median test). (**a**) eRAfam subgroup: the number of swollen joints (68 joint count) was lower in patients with TB in their history when compared with those without this infection (Chi-square = 3.93, df = 1, *p* = 0.05). (**b**) eRAfam subgroup: the serum RF level was lower in patients with TB in their history when compared with those without this infection (Chi-square = 4.73, df = 1, *p* = 0.03). (**c**) eRAfam subgroup: the ESR was lower in patients with pneumonia in their history when compared with those without this infection (Chi-square = 3.81, df = 1, *p* = 0.05). (**d**) eRAaCCP subgroup: the number of swollen joints (68 joint count) was lower in patients with TB in their history when compared with those without this infection (Chi-square = 5.34, df = 1, *p* = 0.02). (**e**) eRAaCCP subgroup: the HAQ value was lower in patients with TB in their history when compared with those without this infection (Chi-square = 9.33, df = 1, *p* = 0.002). (**f**) eRAaCCP subgroup: the ESR was lower in patients with TB in their history when compared with those without this infection (Chi-square = 4.21, df = 1, *p* = 0.04).

In the eRAaCCP high subgroup the number of swollen joints (68 joint count, Chi-square = 5.34, df = 1, *p* = 0.02), the HAQ value (Chi-square = 9.33, df = 1, *p* = 0.002), and the ESR (Chi-square = 4.21, df = 1, *p* = 0.04) were lower if the patient had they had TB in their history (Figure 5).

It should be noted that, for the power analysis, the Spearman rank order correlations results using Cramer’s V effect size of independence, in the cases with a moderately significant correlation (*p* = >0.01 <0.05), it makes sense to increase sample sizes with a 60% chance of obtaining convincing *p*-values (Table 6).

The power analysis in the eRAall cohort also demonstrated impressive chances (with a 60–90% probability), with increasing sample sizes, of identifying a convincing relationship between RA indices and the parameters of acute otitis, skin, and soft tissue infections, exacerbations of chronic otitis, chronic bronchitis, HSV, V-URI, and URIab, as well as the burden of all annual infections (Supplementary Table S1). Moreover, if, considering the relatively large sample sizes of patients with all annual infections burden, V-URI, and URIab, a weak impact of these infections on RA indices can probably be assumed, then, in the case of acute and chronic otitis, chronic bronchitis, HSV, skin, and soft tissue infections, the lack of a significant Spearman correlation with RA indices is definitely due to insufficient sample sizes.

## 4. Discussion

The association of RA with infections has been studied since the end of the XIXth century but from that time it has not been possible to build a clear idea about this link. Our results, conducted in a large cohort of women who developed RA and were analyzed at the early stage of the disease and before disease-modifying antirheumatic drug (DMARD) introduction, confirmed that RA patients have a higher incidence of infections when regarding infections in their anamnesis and at the time of RA onset, with later infections being affected by different factors, such as familial/sporadic history, an elevated immunization against CCP, and a concomitant HSV exacerbation. Associations between infections and RA factors were further reported.

Accordingly, and to better understand the infectious spectrum at the early RA stage (<1 year from onset), we took advantage of the women in Tatarstan’s cohort in order to study the infectious history in the anamnesis as well as infectious events reported in the year preceding eRA onset. The flow chart analysis reported in Figure 1 comprised the cohort description that included healthy controls (n = 398) and eRA patients (n = 195) subdivided into sub-analyses according to their familial/sporadic status and anti-CCP status. Infection incidences from the patient’s history and individual reports (incidence/number/duration) in the previous year were evaluated next. Finally, we looked for associations within the infectious spectrum and RA indices. Results from this work revealed a complex interplay in the women in the Tatarstan cohort between RA and infections that is, in part, under the control of familial status and the presence of elevated levels of anti-CCP antibodies.

An unexpected finding from this study is the impact of having TB in a patient’s history before RA onset on their subsequent annual chronic tonsillitis exacerbations. First of all, the incidence of TB ever in life was found to be remarkably high in all the RA subgroups. Second, TB impacts chronic tonsillitis exacerbations at RA onset, supporting a significant role of TB in RA development. Such an assertion is further supported by the infection cycle of TB, which first enters the pharyngeal lymphoid ring and only then descends into the thorax lymph nodes. Another curious pattern was related to the interplay of pneumonia in the patient’s history before RA onset and chronic bronchitis exacerbation number and duration per year which, to some extent, might be due to some shared immunological and non-immunological mechanisms predisposing to both diseases.

Individual and host genetic factors may be important at RA-onset and such an assertion is supported by our report that eRA patients with a familial history of RA were linked with viral infections (seasonal V-URI, HSV exacerbations, and chronic tonsillitis), while sporadic cases were linked with respiratory tract infections mainly of bacterial etiology, including pneumonia in a patient’s history, URIab, and chronic bronchitis exacerbations. For that, the human leukocyte antigen (HLA) class I and II variants may be incriminated, as recently demonstrated with asymptomatic COVID-19 [16]. To our knowledge, such an analysis has not been conducted in RA between trivial infections, HLA phenotype, and clinical presentation. Future experiments may help to answer this question.

It turned out that annual HSV infection exacerbations increased the incidence and duration of chronic tonsillitis exacerbations. Here, it is appropriate to be reminded of the following. First, about 50% of newborns are infected with the virus while passing through the birth channel [17], so their immune system maturation, including lymphocyte clone selection, takes place in the presence of the virus. Second, HSV DNA is a common finding in biopsy specimens of tonsils in chronic tonsillitis and alone, and, in HSV-microbial associations, causes its exacerbations [18,19]. Additionally, last but not least, tonsils belong to the MALT system, and it is generally accepted that RA at the preclinic stages starts in the mucosa with MALT involvement [1].

The analysis of the dynamics of infections and some immunological parameters in small groups of patients at the early and late stages of RA, as well as relatives of these patients, some of whom developed the disease during observation, presented in our earlier publication, allowed us to formulate the following working hypothesis [4]. Individuals at risk of RA are characterized by a variety of genetically and epigenetically determined defects in immune system factors, some innate receptors and soluble molecules, innate natural killers, and specific anti-herpesviridae cytotoxic CD8 T-lymphocytes, due to inadequate infection susceptibility and immune surveillance, as an example [3]. With that, the functions of some other anti-infectious factors (proinflammatory cytokines, for example, lying on the surface) are redundant. The impossibility of a harmonious, balanced anti-infective response to pathogens according to the principle of reasonable sufficiency leads to frequent and prolonged infections on the one hand and, on the other, to excessive activation of over-functioning factors of the immune system. Due to these over-functioning pro-inflammatory components of the immune system, some people cope with infections, but it is they who develop RA. The more effectively these factors work, the greater the clinical symptoms of RA. This largely speculative scenario for the development of events allows us to, to some extent, explain the paradoxical inverse dependence of RA indices on the frequency and duration of certain infections. Of course, this is just a hypothesis.

Our analysis of the infectious syndrome revealed a possible connection between the disease and the following infections in the Tatarstan cohort of patients with eRA: annual episodes of bacterial infection of upper respiratory tract infection (URIab) as well as exacerbations of HSV infection and chronic tonsillitis. Further, in individuals who later developed RA, encounters with mycobacterium tuberculosis were significantly more likely to result in the development of clinically active infection and antituberculosis therapy than in controls. Moreover, an unexpected and interesting finding was the fact that an episode of TB cured before the onset of RA was linked with chronic tonsillitis exacerbations in a year preceding RA onset and had an effect on eRA disease indices.

To clarify further directions for research, we tried to analyze the possible intersections of the mechanisms of RA pathogenesis and the response of the immune system to these infections.

**Herpesviridae infection susceptibility**. Previously, we analyzed the peculiarities of antiherpes defense in RA [3]. Briefly, HSV1/2 virus infects 60–95% of adults [20]. Given such a high percentage of infection, some authors are even inclined to consider that these viruses should be considered part of the normal microbiome [21]. Thus, the high frequency of HSV reactivation in our cohort is due to insufficient mechanisms that keep viruses persisting in the body in a latent state, and the virus’s tactics to avoid immune detection and establish latency work in a significant portion of the population—up to 80% in human adults for HSV-1 and about 40% for HSV2 [22]. The results of our control cohort showed similar results (the incidence of annual exacerbations 26.6%), while the incidence in the eRAall cohort was reliably increased, being as much as two times higher in the eRAfam subgroup.

Several reasons can be assumed due to the increased incidence of HSV1/2 reactivation in RA. First, reactivation may be provoked by demethylation and histone modification processes, which are excessive in RA [23].

Second, the receptors mediating virus spreading from cell to cell, in particular, herpes virus entry mediator (HVEM) and epidermal growth factor receptor (EGFR), were demonstrated to be overexpressed on most cell types found in RA synovial tissues [24,25]. Serum levels of soluble HVEM are increased in RA as well [26]. Additionally, EGFR gene overexpression in bone marrow-derived mononuclear cells is due to an RA-associated SNP [27,28,29,30].

Third, immune cells recognize viruses by pattern recognition receptors, among which the mannose receptors are considered to be the most important [31,32]. Though some preliminary evidence suggests that mannose receptors may be overexpressed on immune cells of the myeloid lineage present in blood and synovial tissue from RA patients [33], as an important secreted soluble PPR family, the levels of mannose-binding lectin (MBL), an opsonic factor binding to HSV, were found to be decreased in eRA; this is associated with a higher risk of developing early erosive RA and higher levels of IgM RF and CRP [34,35], and the MBL gene is down-regulated more than threefold compared with late-stage RA [36]. The importance of MBL in RA physiopathology is reinforced by the observation of a nucleotide polymorphism (SNP) at the MBL gene associated with RA susceptibility [32,37] and with HSV infection recurrences, possibly due to impaired recognition of the viruses [32].

Fourth, the major deterrents of virus spread limit are type I interferons (IFNs) produced by iNK cells, and IFN-γ produced by macrophages and lymphocytes [38,39,40]. A defective capacity of the immune system to produce IFN-γ in response to viral stimulation was demonstrated, which might be due to the low serum level of IFN-γ and to the recurrence and reactivation of the virus [41,42,43].

Fifth, qualitative and quantitative abnormalities in the iNKT population-minor population of the innate-like T-lymphocytes, which have a specific value in keeping the virus in latency, were demonstrated in early-stage RA. 

The population of the virus-specific CD8+ T-lymphocytes and their cytotoxic activity focused on infected cells or the separate *Herpesviridae* peptide epitopes is decreased as well, both in early- and late-stage RA, in contrast with an abundant and hyperactivated whole population of CD8+ T cells of various specificities [44,45,46].

So, based on the results of the analysis of known facts, it is not surprising that in cohorts of persons at the eRA stage, episodes of HSV1/2 infection reactivation are detected more often than in controls.

**Upper respiratory tract infections.** The development of acute bacterial infections of this localization is mainly due to respiratory viral or other factors affecting the adequate barrier functions of mucous membranes and microbiome structure, as well as to the modulating antibacterial immune response. The following processes are available.

- Local mucosal processes caused by the viral infection—epithelial damage and cell loss, breakdown of mucin by viral neuraminidase, increased receptor availability on epithelial cells due to the promotion of augmented bacterial adherence, inhibition of phagocyte function and neutrophil NET functioning, abnormal expression of antimicrobial host defense peptides, and launching of local immunological processes [47,48,49].
- Microbiome structure modulation– viral mucosal inflammation can create a suitable environment for the growth of opportunistic bacteria and lead to the expression of the receptors and, via cleavage of sialic acid, exposure of receptors to bacteria on the mucosal surface. For example, it was demonstrated that treatment with live attenuated influenza vaccines reverses normal bacterial clearance from the nasopharynx and significantly increases the bacterial carriage densities of Streptococcus pneumoniae and Staphylococcus aureus in mice [50,51].
- Viral inhibition of antibacterial defense. Viral respiratory infection leads to a rapid release of type I interferons to limit viral replication, due to the inhibition of some immune processes and to increase the risk of secondary bacterial infection [49]. In addition to the inhibition of various aspects of phagocytosis, type I IFNs decrease the production of IL-1β and IL-23, which are necessary for the polarization of TH17 cells, and decrease the production of IL-17, correlating with reduced clearance of bacteria [52]. It should be noted here that, in contrast to IFN, gamma type I interferon system responses are increased in RA in correlation with AB production [53]. Following type I IFN production, natural killer cells, but also CD4+ T-helper (TH) cells, CD8+ cytotoxic T cells, and neutrophils, start to release IFN-gamma whose action is not so uniquely inhibitory. It was demonstrated in rodent models of *S pneumoniae* and *S aureus* pneumonia that neutrophil-produced IFN-gamma was produced due to bacterial clearance [54]. However, as mentioned above, in RA there are certain problems with the adequate production of this factor.
- It is now generally accepted that rheumatoid arthritis is triggered by a latent inflammatory process in the mucous membranes, including the upper respiratory tract. From general considerations, it can be assumed that genetically determined features of the functioning of systemic immunity factors probably also determine the peculiarities of the course of local immune processes in the mucous membranes. This little-studied problem is not the subject of discussion in this article.
- In terms of effective neutrophil and macrophage phagocytosis and killing, there are some peculiarities in RA [4,12,55,56].
- The insufficient functioning of the mannose-binding lectin involved in innate immune signaling due to its gene mutation is associated with persistent St. aureus nasopharyngeal carriage [57]. As discussed above, this factor was found to be decreased in eRA due to its gene SNP.
- A critical element of bacterial killing within the phagocytic cell, the antimicrobial components of the intracellular phagocyte granules and, in particular, the cell wall-degrading enzyme lysozyme, are reduced in RA patients and persons at risk [4,58].
- Higher levels of IFN gamma were associated with the successful cleansing of S. aureus from the nose during experimental colonization [59]. IFN gamma deficiency in RA was discussed above.
- The HLA-DRB1*04 RA-associated shared epitope was found to be associated with S. aureus infection in the white population [60].

The special relationships of the immune system with St. aureus were confirmed by a number of authors. Dieperink et al. demonstrated the increased risk of infection due to the RA disease per se regardless of the therapy [61]. The staphylococcal superantigen D gene was found in the synovial fluid and blood of RA patients [62]. Numerous studies should be mentioned, which demonstrate that antigenic determinants of Fc fragments of RF-IgG-epitopes are simultaneously the points of application of Fc-binding proteins of a number of microorganisms, staphylococcus (staphylococcal protein A), streptococci of groups A, C, and G, and herpes simplex virus type I. In other words, RFs might be anti-idiotypic antibodies to the immunoglobulins produced against these pathogens of common infectious processes [63].

**Chronic tonsillitis exacerbations.** First, in chronic tonsillitis provocation, non-infectious factors play a significant role, in particular, smoking and the intake of damaging volatile pollutants (of a domestic and professional nature) [64,65]. The same factors act as RA triggers.

Second, recurrent acute bacterial and viral infections are also important. In chronic tonsillitis, these are adenoviruses, influenza, and parainfluenza viruses, Epstein–Barr viruses, herpes simplex viruses, and enteroviruses of I, II, and V serotype, paving the way for *S. aureus* и *S. pyogenes, Fusobacterium necrophorum*, *Prevotella intermedia*, *Prevotella histicola*, as well as *Mycoplasma pneumoniae,* and *Chlamydia pneumonia* [65].

The loss of barrier functions plays a role as well. In chronic tonsillitis, long-term contact of pathogenic flora with the lacunae epithelial lining is due to epithelial thinning, ulceration, and necrotization, which leads to insufficient barrier function and deepening of the pathological process in the structural elements of the tonsils [66]. The functional activity of the microorganisms, namely, proliferation and RNA synthesis, were revealed not only in epithelial tissue but in connective tissue as well, due to fibroblast activation. Chronic inflammation leads to sclerosis of the parenchyma and a disruption of the cytoarchitectonics of the tonsils, interfering with the normal interplay of the lymphoid and non-lymphoid cells involved in the production of necessary cytokines (the signs of immunodeficiency of the tonsil parenchyma), together with the formation of autoimmune humoral and cellular reactions against the tonsil tissue, and the proliferation of connective tissue antigens. In the local immune organs, the signs of Th2 activity, namely plasmocyte activation, were demonstrated [65].

The scanty information on the features of the functioning of the barrier organs in RA is the subject of a separate review. However, within the framework of the objectives of this discussion, curious details of chronic tonsillitis pathogenesis, in addition to the connections with RA environmental triggers and the role of Th2 reactions in opposing RA, are as follows.

- In chronic tonsillitis, low levels of mannose-binding lectin play a role (in RA, as discussed above) [67].
- A decrease in the local level of lysozyme in both infections in RA-discussed above) [65,67].
- Impaired phagocytosis of bacteria [68], (in RA, as discussed above).
- Stimulation of TGF-β1 (transforming growth factor β1), local production by microorganisms, and modulation of local immune Th17 reaction [69]; increased expression of this factor is known to play a role in RA [70,71,72].

**TB ever in life.** Almost all publications on the problem of RA and TB are studies of the risk of developing infection against the background of modern biological therapy. Meanwhile, we demonstrated that persons predisposed to RA have the honor of belonging to the minority of the population (5–10%) that develop primary active TB disease, with clinical symptoms ever in life, while the majority of infected persons show no disease symptoms but develop an effective acquired immune response and are referred to as having a latent infection [73]. It must be noted that our data are consistent with the study results of Spanish authors who analyzed the data of the National Network of Epidemiological Surveillance reports from 1990 to 2000 and found a 4-fold increased risk of TB infection in patients diagnosed with RA [74].

Analysis of the literature data revealed a number of weak links in the immune system in RA, which could lead to insufficient immune surveillance of infection.

The first step is the efficient uptake and destruction of MB by macrophages [75]. We and other researchers demonstrated the abnormalities of phagocyte functioning, namely, phagosome formation, in RA and persons at risk [55].

Second, as well as in the case of herpesviruses, reception of numerous MB ligands by macrophages is carried out by pattern recognition receptors, particularly the mannose receptor and mannose-binding lectin [76], the latter of which was found to be deficient in RA, as was discussed above.

Third, adequate functioning of innate lymphocytes and natural killer (NK) cells possessing potent cytolytic capacity is also known to be impaired in RA [76], as was discussed above.

Fourth, the microbiome was relatively recently recognized as a new player. Although the study of the relationship between the microbiome of different localization and TB is only gaining momentum, evidence has been obtained of the influence of the microbiome of the nasopharynx, oral cavity, lungs, and the intestine (due to the functioning of the intestinal–lung axis) on the anti-tuberculosis response [77]. For example, latent TB patients that possessed serum anti-*H. pylori* antibodies demonstrated higher TB antigen-specific Th1 responses and IFN-γ production and were less likely to develop active TB disease compared to *H. pylori* seronegative individuals [78]. Butyrate produced by *Prevotella* inhibited the mycobacterial antigen-specific IL-17 and IFN-γ responses and increased lung MB-specific regulatory T cells [79]. It must be noted that both mechanisms of modulating the anti-TB immune response affect RA development in the opposite direction, Th1 stimulation by *H. pylori* might trigger RA, while the microbial HCFA inhibitory effect can counteract the disease development. At the same time, the genus *Prevotella* is a well-known trigger of ACCP production.

In the same way, the opposite impact of the Natural Resistance Associated Macrophage Protein (Nramp1) phagosomal bivalent cation transporter Nramp-1 functioning has been observed in these two diseases. TB-associated gene mutations of this factor are due to the weakening of the response of macrophages to infection and increasing TB susceptibility [80,81]. RA-associated polymorphisms of this gene cause the excessive activity of this factor and contribute to the aggravation of pro-inflammatory mechanisms [82].

After TB episodes ever in life, MB persists mostly in the lung macrophages in a quiescent or latent state. Maintaining this state unconditionally is a process (immune surveillance) whose effectiveness is determined by the permanent activity of the host’s immune system [83], namely, macrophages, Th17 cells with a long-lived effector memory phenotype and memory Th17 cells, polyfunctional T cells, non-conventional γδ T cells, mucosa-associated invariant T (MAIT) cells, and natural killers (NK), with the possible participation of antibody-producing β-lymphocytes. Most of these cells are active cytokine producers, with IFN gamma, IL-17, TNF, IL-1β, Il-22, Il-12, and Il-2, as well as Thelper2 (Th2)-associated cytokine being the most important [84]. Effective surveillance is due to the delicate balance of these factors. In addition, according to the currently scarce data, post-TB infection and therapy leave a long mark on the structure of the respiratory tract microbiome [85].

Active immune surveillance of herpesviridae infection (in particular varicella zoster virus chickenpox etiological factor, followed by neurons for long-term residence and reactivation, with the clinical symptoms of herpes zoster) is carried out by macrophages producing IFNs, TNF-α, and IL-6, and lymphocytes producing IFN-γ [38,39,40]. Activated CD4+ and CD8+ T cells play a pivotal role in clearing the primary infection [40,86]. Nonspecific and specific CD8+ T cells infiltrate and persist within virus-infected cells, with specific CD8+ T-lymphocytes being the prevailing ones, expressing a late effector memory phenotype and being activated by stimulation from the infected cells [87,88,89]. The role of B cells in the immune response to herpesviridae is to present antigens and secrete cytokines [90,91,92]. It is reasonable to specify that, even when latent, the herpes viral genome is not completely silenced, and some viral proteins are produced. In the case of EBV, it was demonstrated that it exhibits three different latency programs, each comprising a limited and distinct set of viral proteins and RNAs [21].

So, despite being latent, the infection is still a challenge for the host immune system. In a host that is predisposed to RA with imbalanced immune reactions, two consequences may arise: (i) insufficiently effective immune surveillance may cause latency with greater pathogen activity; (ii) there may be a tendency to uncontrolled pro-inflammatory reactions and insufficient inhibitory mechanisms and constant background cell stimulation may ultimately be one of the triggers for the development of the disease.

The above considerations are also valid for another member of herpesviridae alpha subfamily—HSV1/2. Despite the fact that we conducted an examination in the absence of signs of this infection, as well as of other commonplace infections, the impact of viral reactivation during the last year on clinical and laboratory parameters of the eRA cohorts was found. Previously, we demonstrated the link between the increased granulocyte spontaneous and stimulated ROS production in eRA on one hand, and the ROS production indexes with RA activity indexes [12] on the other. V-URI infectious episodes in the last year were linked with increased spontaneous ROS production as well. Our data are supported by the well-known fact that ROS are known as the basic pathogen molecules in viral infections [21,93]. We also demonstrated HSV DNA in blood PBMC and abnormalities of antibody production in RA in connection with its activity were found [5].

So, the analysis revealed certain prerequisites for an increased susceptibility to certain identified infections in individuals prone to developing RA.

On the other hand, some features of innate and adaptive immunity that are characteristic of RA may contribute to the provocation of disease by these infections. This publication does not aim to analyze the provoking mechanisms in detail, but briefly, it can be assumed that the following features of innate and adaptive immunity that are characteristic of RA may contribute to the provocation of disease by these infections.

- The inflammatory process in the mucous membranes of barrier organs leads to disruption of the microbiome structure and increased mucosal permeability to inflammatory factors and pathogens, as well as to the appearance of portions of post-translationally modified proteins (citrullinated, glycosylated) and the development of an adaptive immune response to them.
- Provocation by infectious pathogens sets in motion the powerful pro-inflammatory potential of the immune system due to polymorphisms in genes of the NFkB and Jak/Stat signaling pathways, cytokines, and Toll-like receptor genes, and disruption of the formation of inflammasomes [3,94].
- Imperfect control of lymphocyte activity, due to SNPs of PTPN22, CTLA-4, BTLA, and some other elements of the immune response [95,96], makes it difficult to stop immune activity that is no longer needed after recovery from infection. It is also necessary to add the Treg/Th17 balance and imperfect suppressor activity of Treg cells in RA, due, in particular, to the modulation of TNF alpha [97,98,99]. According to some data, suppression of Treg cell activity and a shift in Treg/Th17 balance is fraught with the breakdown of self-tolerance, leading to the progression of RA which might be due to recurrent HSV1/2 infection [100].

In terms of the limits of this study, the authors understand that information about specific trivial infections carried during the year, and the number and duration of these infections, is subjective. However, a significant number of publications are based on the results of analysis of data obtained by quantitative interviews (175 publications in PubMed when searching for the keywords “rheumatoid arthritis” and “quantitative interviews”), some of which are based on online or phone surveys and information self-reported by patients [101,102,103,104,105]. In our study, information about annual infections was collected face-to-face from year to year (up to 15 or more years of monitoring) by a qualified rheumatologist with extensive experience as a general practitioner and included the active use of information from medical records (about 30–40% of annual acute infections, 100% of exacerbation of chronic infections other than HSV, and 100% of pneumonia, TB, and contraction of sexual infections ever in life). We tried to balance the subjective component with a critical approach to information collecting and a multi-layered statistical analysis.

## Figures and Tables

**Table 1:** Characteristic of the cohorts ^#^.

	Controls	eRA				
		All	Fam	Spo	* aCCP > 3 ULN	* aCCP Neg/Low
Age, years, median, IQR	48 (33)	50.5 (50.5)	49 (49)	51 (51)	52 (20)	49 (20)
Gender: female/male (female %)	382/16 (96)	192/3 (98.5)	89/2 (97.8)	70/1 (98.6)	65/2 (97.0)	41/0 (100)
Fam/Spor (%)		91/71 (56.2)			43/24 (64.2)	23/18 (56.1)
DAS28-ESR, score median (IQR), high/moderate/low, %		5.2 (45)	4.7 (48)	5.5 (44)	5.5 (47) 54.5/38.2/7.3	5.1 (34) 48.6/48.6/2.8
HAQ, score, median (IQR)		1.38 (33)	1.16 (25)	1.88 (38)	1.2 (100)	1.88 (74)
ESR, mm/hour median (IQR)		34 (23)	32 (28)	33 (22)	34 (24)	30 (25)
CRP, mg/mL median (IQR)		8.57 (20)	10.0 (24)	7.0 (14.5)	12.0 (25)	7.0 (10.5)
RF median (IQR)		56.8 (76.5)	41.6 (55.5)	60.0 (65)	64.0 (24)	32.0 (57)
RF pos/neg (%)		109/29 (79%)	51/20 (71.8)	39/22 (63.9)	53/14 (79.1)	28/13 (68.3)
aCCP median (IQR)		116.1 (238.5)	139.1 (401)	113.0 (397.5)	506.0 (645)	
aCCP pos/neg (%)		123/27 (82.0)	79/3 (96.3)	58/5 (92.1)		

In 27 cases, heredity information was incomplete. * aCCP neg/low—1–3 cut-off level, aCCP > 3 ULN—≥3x cut-off level. ^#^ There was no reliable difference in demographic parameters in all the cohorts and in the laboratory and complex activity indices in RA subgroups when analyzed using the Mann–Whitney and Chi2 tests.

**Table 2:** Criteria used in the questionnaire to establish infections.

Infection	Criteria of the Diagnosis	Questionnaire			Clinical Confirmation
		Yes/No	Number	Episode Duration	Yes/No
No infections during last year	Declared by the individual, no clinical manifestation of any infection within the last year.	Yes	-	-	-
Acute upper respiratory tract infection symptoms (URI) of multiple and unspecified site (ICD10 *: X J06)	URI symptoms: catarrhal phenomena; not accompanied by itchy skin rashes in the form of urticaria, angioedema; gradually developing with a prodromal period in the form of increasing symptoms of malaise (intoxication-like feelings, low-grade fever, headache, myalgia, arthralgia); lasting 3–14 days (in cases not complicated by secondary bacterial infections); developed as a result of contact with an infected persons, as well as after general and local cooling, overheating, or emotional/mental and physical stress (but not after known contact with the individual allergens or potential allergens or while receiving either of the drug); treatment carried out with antiviral and, optionally, antipyretic drugs (but not antihistamines).	Yes	Yes	Yes	No
Herpes simplex type I/II virus (HSV) infection exacerbations (B00, A60)	HSV infection was evaluated by questioning about typical clinical manifestations (blisters mainly on the lip and nose mucosa after hypothermia events, lack of sleep, mental stress, the effect of local antiviral therapy). In solitary cases of herpetic stomatitis or keratitis, or atypical dermal or mucosal localization of the blisters, the diagnosis was verified by the ear, nose, and throat (ENT) doctor, dentist, dermatologist, or oculist based on the clinical, laboratory (serology), and instrumental examination. In case of genital infections, the frequency and duration of HSV exacerbation events was assessed by a gynecologist.	Yes	Yes	Yes	Yes except blister
Herpes zoster (B02)	In all cases, the diagnosis was made by a general practitioner based on the clinical, laboratory (serology), and instrumental examination, and further confirmed by a specialist in infectious diseases.	Yes	-	-	Yes
Tonsillitis/sinusitis/bronchitis/otitis (acute and exacerbations of the chronic) (H60–H95, J00–J05, J42, J20)	The diagnosis was made by a general practitioner who treated the patient in uncomplicated cases. With a more severe/protracted episode, and in all cases of exacerbation of the chronic infectious focus, the diagnosis was verified by an ENT doctor. The use of antihistaminic drugs was an exclusion criterion.	Yes	Yes	Yes	Yes
Pneumonia ever in life (before RA onset) (J18)	In all cases, the diagnosis was made by a general practitioner and confirmed in the therapeutic department of a regional hospital for treatment.	Yes	Yes	Yes	Yes
Upper and lower urinary tract infections (acute and exacerbations of the chronic one) (N30, N34.0–34.2, N10, N11)	In all cases, the diagnosis was made by a general practitioner and was confirmed by the urologist who has examined and treated the patient.	Yes	Yes	Yes	Yes
Oral and odontical infections (K05)	Stomatitis; periodontitis was assessed by a dentist.	Yes	Yes	Yes	Yes
Skin and soft tissue infections (L00–L99)	Pyoderma, streptoderma, erysipelas, or carbuncle/furuncle was assessed by a dermatologist or a surgeon (in case of surgical opening of carbuncle/furuncle).	Yes	Yes	Yes	Yes
Tuberculosis in anamnesis (A15–A19)	TB infection in anamnesis verified by TB specialist based on the clinical, laboratory (serology, Quantiferon assay), and instrumental examination.	Yes	-	-	Yes
Chlamydia, mycoplasma, ureaplasma infection at pregnancy (A60, A63.8, A49.3)	As a laboratory diagnosis of chlamydia, mycoplasma, ureaplasma infection is mandatory for all pregnant women and when planning pregnancy, we also considered asymptomatic carriage of these infections in the laboratory test data. Infectious episodes were assessed by appropriate specialists (gynecologists, ENT doctors, pulmonologists).	Yes	-	-	Yes

* Disease codes according to the International Classification of Diseases (ICD10).

**Table 3:** Incidence of infections in the anamnesis of eRA patients as compared to healthy controls, * n (yes/no, %), Chi2 without Yates’ correction, one-tailed ^ *p*-value.

Infections	Controls	eRAall	eRAfam	eRAspo	^&^ aCCP > 3 ULN eRA	^&^ aCCP neg/low eRA
Ever in life						
Pneumonia	54/336 (13.8)	31/94 (27.8)	13/58 (18.3)	12/30 (28.6)	9/46 (16.4)	9/18 (33.3)
			eRAfam vs. spo *p* = 0.1 ** 1-β = 0.2 (80%)		aCCP high vs. neg/low *p* = **0.04** **1-β** = 0.3 (70%)	
	vs. Controls *p* =	**0.002** **1-β** = 0.7 (30%)	0.2 **1-β** = 0.1 (90%)	**0.006** **1-β** = 0.6 (40%)	0.3 **1-β** = 0.06 (94%)	**0.003** **1-β** = 0.7 (30%)
Herpes zoster	5/391 (1.3)	5/163 (3.0)	1/78 (1.3)	1/55 (1.8)	1/58 (1.7)	1/33 (2.9)
			eRAfam vs. spo *p* = 0.4 **1-β** = 0.04 (96%)		aCCP high vs. neg/low *p* = 0.3 **1-β** = 0.06 (94%)	
	vs. Controls *p* =	0.08 **1-β** = 0.2 (80%)	0.5 **1-β** = 0.03 (97%)	0.4 **1-β** = 0.04 (96%)	0.4 **1-β** = 0.04 (96%)	0.2 **1-β** = 0.1 (90%)
Tuberculosis	1/354 (0.28)	15/80 (15.8)	**12/45 (21.1)**	**3/31 (8.8)**	7/35 (16.7)	3/18 (14.3)
			eRAfam vs. spo *p* = 0.06 **1-β** = 0.2 (80%)		aCCP high vs. neg/low *p* = 0.4 **1-β** = 0.04 (96%)	
	vs. Controls *p* =	**0.0001** **1-β** = 0.9 (1%)	**0.0001** **1-β** = 0.9 (1%)	**0.0001** **1-β** = 0.9 (1%)	**0.0001** **1-β** = 0.9 (1%)	**0.0001** **1-β** = 0.9 (1%)
Carriage of *Chlamydia*, *Mycoplasma*, and *Ureoplasma* sp.	14/65 (17.7)	19/38 (33.3)	11/23 (32.3)	8/15 (34.7)	12/22 (35.3)	2/12 (14.3)
			eRAfam vs. spo *p* = 0.4 **1-β** = 0.04 (96%)		aCCP high vs. neg/low *p* = 0.07 **1-β** = 0.2 (80%)	
	vs. Controls *p* =	**0.02** **1-β** = 0.4 (96%)	**0.04** **1-β** = 0.3 (70%)	**0.04** **1-β** = 0.3 (70%)	**0.02** **1-β** = 0.4 (60%)	*p* = 0.4 **1-β** = 0.04 (96%)

* represented n of infection positive cases/n of infection negative cases (% of positive cases in a cohort). ^ *p* ≤ 0.01—reliable difference (red); *p* ≥ 0.05—possible difference (hypothesis is notable and needs more study, blue). ^&^ aCCP neg/low—1–3 cut-off level, aCCP > 3 ULN—≥3× cut-off level. ** 1-β—power of independence criteria (Cramer’s V effect size). In parentheses—the probability (%) that as the sizes of compared cohorts increase, a reliable difference in the indicators will appear or increase.

**Table 4:** Incidence of infections reported in the previous year of eRA patients as compared to healthy controls, number (yes/no, %), Chi-square without Yates’ correction, one-tailed ^^^ *p*-value.

Infections	Controls	eRAall	eRAfam	eRAspo	^&^ aCCP > 3 ULN eRA	^&^ aCCP Neg/Low eRA
All infections (there were infections/year or not one infection/year)	337/61 (84.7)	184/11 (94.4)	89/2 (97.8)	66/5 (93.0)	63/4 (94.0)	39/2 (95.1)
			*p* = 0.1 * 1-β = 0.2		*p* = 0.4 **1-β** = 0.04	
	vs. Controls *p* =	**0.0003** **1-β** = 0.9 (10%)	**0.0004** **1-β** = 0.8 (20%)	**0.02** **1-β** = 0.4 (60%)	**0.02** **1-β** = 0.4 (60%)	**0.03** **1-β** = 0.3 (70%)
V-URI	229/169 (57.5)	106/89 (54,4)	57/34 (62.6)	39/32 (54.9)	42/25 (62.6)	27/14 (65.9)
			*p* = 0.2 **1-β** = 0.1 (90%)		*p* = 0.4 **1-β** = 0.04 (96%)	
	vs. Controls *p* =	0.2 **1-β** = 0.1 (90%)	0.2 **1-β** = 0.1 (90%)	0.1 **1-β** = 0.2 (80%)	0.2 **1-β** = 0.1 (90%)	0.2 **1-β** = 0.1 (90%)
HSV1/2 infection exacerbation	106/292 (26.6)	76/119 (39.0)	41/50 (54.9)	28/43 (39.4)	33/34 (49.3)	17/24 (41.5)
			0.2 **1-β** = 0.1 (90%)		*p* = 0.2 **1-β** = 0.1 (90%)	
	vs. Controls *p* =	**0.001** **1-β** = 0.8 (20%)	**0.0003** **1-β** = 0.9 (10%)	**0.01** **1-β** = 0.5 (50%)	**0.0001** **1-β** = 0.9 (10%)	**0.02** **1-β** = 0.4 (60%)
URI with antibiotics	52/346 (13.1)	41/154 (21.0)	13/78 (14.3)	21/50 (29.5)	14/53 (20.9)	8/33 (19.5)
			*p* = **0.009** **1-β** = 0.5 (50%)		*p* = 0.4 **1-β** = 0.04 (96%)	
	vs. Controls *p* =	**0.006** **1-β** = 0.6 (40%)	0.3 **1-β** = 0.06 (94%)	**0.0002** **1-β** = 0.9 (10%)	**0.04** **1-β** = 0.3 (70%)	0.1 **1-β** = 0.2 (80%)
Acute otitis	3/395 (0.75)	8/187 (4.1)	4/87 (4.4)	3/68 (4.2)	2/65 (3.0)	5/36 (12.2)
			*p* = 0.5 **1-β** = 0.03 (97%)		***p* = 0.03** **1-β** = 0.3 (70%)	
	vs. Controls *p* =	**0.002** **1-β** = 0.7 (30%)	**0.004** **1-β** = 0.6 (40%)	**0.008** **1-β** = 0.5 (50%)	**0.05** **1-β** = 0.3 (70%)	**0.0001** **1-β** = 0.9 (10%)
Chronic otitis exacerbation	0/398 (0)	5/190 (2.6)	4/87 (4.4)	0/71 (0)	1/66 (1.5)	0/41 (0.0)
			***p* = 0.03** **1-β** = 0.3 (97%)		*p* = 0.2 **1-β** = 0.1 (90%)	
	vs. Controls *p* =	**0.0007** **1-β** = 0.8 (20%)	**0.0001** **1-β** = 0.9 (10%)	Z **1-β** = 0.01 (99%)	**0.007** **1-β** = 0.5 (50%)	Z **1-β** = 0.01 (99%)
Acute tonsillitis	46/352 (11.6)	34/161 (17.4)	15/76 (16.5)	13/58 (18.3)	7/60 (10.4)	8/33 (19.5)
			*p* = 0.4 **1-β** = 0.04 (96%)		*p* = 0.09 **1-β** = 0.2 (80%)	
	vs. Controls *p* =	**0.02** **1-β** = 0.4 (60%)	**0.05** **1-β** = 0.3 (70%)	0.06 **1-β** = 0.2 (80%)	0.4 **1-β** = 0.04 (96%)	0.07 **1-β** = 0.2 (80%)
Chronic tonsillitis exacerbation	17/381 (4.3)	34/161 (17.4)	19/72 (20.9)	11/60 (15.5)	11/56 (16.4)	5/36 (12.1)
			*p* = 0.1 **1-β** = 0.2 (80%)		*p* = 0.3 **1-β** = 0.06 (94%)	
	vs. Controls *p* =	**0.0001** **1-β** = 0.9 (10%)	**0.0001** **1-β** = 0.9 (10%)	**0.0001** **1-β** = 0.9 (10%)	**0.0001** **1-β** = 0.9 (10%)	**0.01** **1-β** = 0.5 (50%)
Acute sinusitis	10/388 (2.6)	8/187 (4.1)	5/86 (5.5)	1/70 (1.4)	2/65 (3.0)	2/39 (4.9)
			*p* = 0.09 **1-β** = 0.2 (80%)		*p* = 0.3 **1-β** = 0.06 (94%)	
	vs. Controls *p* =	0.1 **1-β** = 0.2 (80%)	0.07 **1-β** = 0.2 (80%)	0.3 **1-β** = 0.6 (40%)	0.4 **1-β** = 0.04 (96%)	0.2 **1-β** = 0.1 (90%)
Chronic sinusitis exacerbation	7/391 (1.7)	10/185 (5.1)	7/84 (7.7)	2/69 (2.8)	4/63 (6.0)	0/41 (0.0)
			*p* = 0.09 **1-β** = 0.2 (80%)		*p* = 0.06 **1-β** = 0.2 (80%)	
	vs. Controls *p* =	**0.01** **1-β** = 0.5 (50%)	**0.001** **1-β** = 0.8 (20%)	0.2 **1-β** = 0.1 (90%)	**0.02** **1-β** = 0.4 (60%)	0.2 **1-β** = 0.1 (90%)
Acute bronchitis	16/382 (4.0)	13/182 (6.7)	5/86 (5.5)	5/66 (7.0)	1/66 (1.5)	2/39 (4.9)
			*p* = 0.3 **1-β** = 0.06 (96%)		*p* = 0.1 **1-β** = 0.2 (80%)	
	vs. Controls *p* =	0.08 **1-β** = 0.2 (80%)	0.3 **1-β** = 0.06 (96%)	0.1 **1-β** = 0.2 (80%)	0.2 **1-β** = 0.2 (80%)	0.4 **1-β** = 0.4 (60%)
Chronic bronchitis exacerbation	8/390 (2.0)	17/178 (8.7)	6/85 (6.6)	7/64 (9.9)	3/64 (4.5)	3/38 (7.3)
			*p* = 0.2 **1-β** = 0.1 (90%)		*p* = 0.3 **1-β** = 0.06 (94%)	
	vs. Controls *p* =	**0.0001** **1-β** = 0.9 (10%)	**0.009** **1-β** = 0.5 (50%)	**0.0003** **1-β** = 0.9 (10%)	0.1 **1-β** = 0.2 (80%)	**0.02** **1-β** = 0.4 (60%)
Urinary tract infection	8/390 (2.0)	10/185 (5.1)	1/90 (1.1)	7/64 (9.9)	4/63 (6.0)	3/38 (7.3)
			***p* = 0.005** **1-β** = 0.6 (40%)		*p* = 0.4 **1-β** = 0.04 (96%)	
	vs. Controls *p* =	**0.02** **1-β** = 0.4 (60%)	0.3 **1-β** = 0.06 (94%)	**0.0003** **1-β** = 0.9 (10%)	**0.03** **1-β** = 0.3 (70%)	**0.02** **1-β** = 0.4 (60%)
Oral infection	1/397 (0.25)	5/190 (1.9)	2/89 (2.2)	3/68 (4.2)	1/66 (1.5)	2/39 (4.9)
			*p* = 0.2 **1-β** = 0.1 (90%)		*p* = 0.1 **1-β** = 0.2 (80%)	
	vs. Controls *p* =	**0.004** **1-β** = 0.6 (40%)	**0.02** **1-β** = 0.4 (60%)	**0.0004** **1-β** = 0.8 (20%)	0.08 **1-β** = 0.01 (99%)	**0.0003** **1-β** = 0.9 (10%)
Skin infection	8/390 (2.0)	19/176 (9.7)	11/80(12.1)	5/66 (7.0)	12/55 (17.9)	0/41 (0.0)
			*p* = 0.1 **1-β** = 0.2 (80%)		***p* = 0.002** **1-β** = 0.7 (30%)	
	vs. Controls *p* =	**0.0001** **1-β** = 0.9 (10%)	**0.0001** **1-β** = 0.9 (10%)	**0.009** **1-β** = 0.5 (50%)	**0.0001** **1-β** = 0.9 (10%)	*p* = 0.2 **1-β** = 0.1 (90%)
Association of HSV reactivation and tonsillitis and respiratory infections						
HSV + V-URI	69/329 (17.3)	44/150 (22.7)	23/68 (25.3)	16/55 (22.5)	22/45 (32.8)	11/6 (64.7)
			*p* = 0.3 **1-β** = 0.06 (94%)		***p* = 0.008** **1-β** = 0.5 (50%)	
	vs. Controls *p* =	0.06 **1-β** = 0.2 (80%)	**0.04** **1-β** = 0.3 (70%)	0.3 **1-β** = 0.06 (94%)	**0.003** **1-β** = 0.5 (50%)	**0.0001** **1-β** = 0.9 (10%)
HSV + B-URI antibiotics	15/383 (3.7)	15/179 (7.7)	8/83 (8.8)	5/66 (7.0)	7/60 (10.4)	4/13 (23.5)
			*p* = 0.3 **1-β** = 0.06 (94%)		*p* = 0.08 **1-β** = 0.2 (80%)	
	vs. Controls *p* =	**0.02** **1-β** = 0.4 (60%)	**0.02** **1-β** = 0.4 (60%)	0.1 **1-β** = 0.2 (80%)	**0.009** **1-β** = 0.5 (50%)	**0.0001** **1-β** = 0.9 (10%)
HSV + Acute tonsillitis	15/383 (3.7)	15/179 (7.7)	6/85 (6.6)	7/64 (9.9)	3/64 (4.5)	7/10 (41.2)
			*p* = 0.2 **1-β** = 0.1 (90%)		***p* = 0.0001** **1-β** = 0.9 (10%)	
	vs. Controls *p* =	**0.02** **1-β** = 0.4 (60%)	0.1 **1-β** = 0.2 (80%)	**0.01** **1-β** = 0.5 (50%)	0.4 **1-β** = 0.04 (96%)	***p* = 0.0001** **1-β** = 0.9 (10%)
HSV + Chronic tonsillitis	5/393 (1.6)	8/186 (4.4)	6/85 (6.6)	4/67 (5.6)	0/67 (0.0)	1/16 (5.9)
			*p* = 0.4 **1-β** = 0.05 (95%)		***p* = 0.02** **1-β** = 0.4 (60%)	
	vs. Controls *p* =	**0.01** **1-β** = 0.5 (50%)	**0.001** **1-β** = 0.8 (20%)	**0.007** **1-β** = 0.5 (50%)	0.2 **1-β** = 0.1 (90%)	*p* = 0.06 **1-β** = 0.2 (80%)
HSV + Chronic bronchitis	1/397 (0.25)	4/190 (2.1)	2/89 (2.2)	2/69 (2.8)	0/67 (0.0)	2/15 (11.8)
			*p* = 0.4 **1-β** = 0.04 (96%)		***p* = 0.002** **1-β** = 0.7 (30%)	
	vs. Controls *p* =	**0.01** **1-β** = 0.5 (50%)	**0.02** **1-β** = 0.4 (60%)	**0.006** **1-β** = 0.6 (40%)	0.3 **1-β** = 0.06 (94%)	***p* = 0.0001** **1-β** = 0.9 (10%)

^ *p* ≤ 0.01—reliable difference (red); *p* ≥ 0.05—possible difference (hypothesis is notable and needs more study, blue). ^&^ aCCP neg/low—1–3 cut-off level, aCCP > 3 ULN—≥3x cut-off level. * 1-β—power of independence criteria (Cramer’s V effect size). In parentheses—the probability (%) that, as the sizes of compared cohorts increase, a reliable difference in the indicators will appear or increase.

**Table 5:** Parameters of annual infections, Kruskal–Wallis median test.

Infection		Controls (1)	eRAall (2)	eRAfam (3)	eRAspo (4)	^&^ aCCP > 3 ULN eRA (5)	^&^ aCCP low/neg eRA (6)
All infections	Number/year median, quartile 1–4 (n)	2.0; 1.0–4.0 (398)	5.0; 3.0–8.0 (195)	5.0; 3.0–8.0 (91)	4.5; 3.0–10.0 (71)	5.0; 3.0–10.0 (67)	8.8; 7–12.3 (41)
				*p* = 0.2		*p* = 0.4	
		vs. Controls *p* =	^^^ 0.000	0.0000	0.0000	0.0000	0.00000
		* 1-β_1–6_ =	1.0 (0%)				
	Episode duration median, quartile 1–4 (n)	6.2; 3.0–7.0 (398)	9.2; 6.5–13.3 (195)	9.0; 7.0–11.7 (91)	8.7; 5.7–13.2 (71)	8.8; 6.5–12.0 (67)	13.6; 3–10 (41)
				*p* = 0.9		*p* = 0.4	
		vs. Controls *p* =	0.000	0.0000	0.0000	0.0000	0.00000
		**1-β_1–6_** =	1.0 (0%)				
V-URI	Number/year median, quartile 1–4 (n)	2.0; 1.0–2.0 (229)	2.0; 1.0–4.0 (106)	3.0; 2.0–4.0 (53)	2.0; 2.0–3.5 (39)	3.0; 1.0–4.0 (42)	3.0; 2.0–4.0 (27)
				*p* = 0.1		*p* = 0.3	
		vs. Controls *p* =	0.0000	0.0000	0.0000	0.001	0.002
		**1-β_1–6_** =	0.6 (40%)				
	Episode duration median, quartile 1–4 (n)	7.0; 3.0–7.0 (229)	7.0; 3.5–7.0 (106)	7.0; 3.0–7.0 (53)	7.0; 4.5–7.0 (39)	7.0; 4.0–9.0 (42)	7.0; 4.0–9.5 (27)
				*p* = 0.9		*p* = 0.8	
		vs. Controls *p* =	0.6	0.5	0.7	0.3	0.07
		**1-β_1–6_** =	1.0 (0%)				
HSV 1/2 exacerbation	Number/year median, quartile 1–4 (n)	2.0; 1.0–3.0 (106)	2.0; 1.0–3.0 (76)	2.0; 1.0–4.0 (41)	2.0; 1.0–2.2 (28)	2.0; 1.0–4.0 (33)	3.0; 1.0–4.0 (17)
				*p* = 0.2		*p* = 0.2	
		vs. Controls *p* =	0.5	0.8	0.2	0.5	0.06
		**1-β_1–6_** =	0.5 (50%)				
	Episode duration median, quartile 1–4 (n)	7.0; 3.0–7.0 (106)	7.0; 4.5–10.0 (76)	7.0; 4.0–10.0 (41)	7.0; 3.5–10.0 (20)	7.0; 5.0–10.0 (33)	7.0; 7.0–10.0 (17)
				*p* = 0.6		*p* = 0.3	
		vs. Controls *p* =	0.09	0.1	0.1	0.01	0.01
		**1-β_1–6_** =	1.0 (0%)				
URI with antibiotics	Number/year median, quartile 1–4 (n)	2.0; 1.0–2.0 (52)	2.0; 1.0–4.0 (41)	2.0; 1.0–4.0 (14)	1.0;1.0–4.0 (21)	2.0; 1–12 (14)	1.0; 1.0–6.0 (8)
				*p* = 0.6		*p* = 0.3	
		vs. Controls *p* =	0.5	**0.02**	0.3	0.1	0.9
		**1-β_1–6_** =	0.3 (70%)				
	Episode duration median, quartile 1–4 (n)	7.0; 4.0–12.0 (52)	14.0; 10.0–27.0 (41)	14.0; 7.0–21.0 (14)	14.0; 10.0–45.0 (21)	10.0; 7–270 (14)	14.0; 11.0–30.0 (8)
				*p* = 0.8		*p* = 0.4	
		vs. Controls *p* =	0.0001	**0.03**	0.004	0.2	0.002
		**1-β_1–6_** =	1.0 (0%)				
Acute tonsillitis	Number/year median, quartile 1–4 (n)	2.0; 1.0–3.0 (46)	2.0; 1.0–3.0 (34)	3.0; 1.0–4.0 (15)	2.0; 1.0–3.0 (13)	4.0; 2–12 (7)	7.0; 5.5–12.5 (8)
				*p* = 0.4		*p* = 0.2	
		vs. Controls *p* =	**0.05**	**0.05**	0.1	**0.02**	0.2
		**1-β_1–6_** =	0.7 (30%)				
	Episode duration median, quartile 1–4 (n)	5.5; 3.0–7.0 (46)	7.0; 7.0–14.0 (34)	11.0; 7.0–14.0 (15)	7.0; 4.0–10.0 (13)	7.0; 5.5–14 (7)	2.5; 1.5–8.5 (8)
				*p* = 0.3		*p* = 0.7	
		vs. Controls *p* =	0.002	0.0006	0.1	0.2	0.7
		**1-β_1–6_** =	1.0 (0%)				
Chronic tonsillitis exacerbation	Number/year median, quartile 1–4 (n)	4.0; 3.0–12.0 (17)	6.0; 3.0–9.0 (34)	5.0; 1.0–6.0 (19)	6.0; 3.0–12.0 (11)	7.0; 5,5–14(11)	6.0; 4.0–12.0 (^#^ 5)
				*p* = 0.2		*p* = 0.5	
		vs. Controls *p* =	0.8	0.7	0.2	0.3	0.7
		**1-β_1–6_** =	1.0 (0%)				
	Episode duration median, quartile 1–4 (n)	7.0; 7.0–10.0 (17)	10.0; 7.0–14.0 (34)	10.0; 8.0–14.0 (19)	8.5; 4.7–14.0 (11)	8.0; 4.7–14(11)	12.0; 8.0–12.0 (^#^ 5)
				*p* = 0.6		*p* = 0.6	
		vs. Controls *p* =	**0.04**	0.06	0.7	0.7	0.9
		**1-β_1–6_** =	1.0 (0%)				
Acute bronchitis	Number/year median, quartile 1–4 (n)	2.0; 1.0–2.0 (16)	2.0; 1.0–3.0 (13)	3.0; 2.0–4.0 (^#^ 5)	2.0; 1.0–3.0 (^#^ 5)	n = 1	n = 2
		vs. Controls *p* =	0.1	**0.03**	0.2		
		**1-β**_1–4_	0.3 (70%)				
	Episode duration median, quartile 1–4 (n)	12.5; 7.8–14.0 (16)	14.0; 14.0–18.0(13)	17.0; 14.0–18.0 (^#^ 5)	14.0; 11.0–14.7 (^#^ 5)		
		vs. Controls *p* =	**0.04**	**0.03**	0.2		
		**1-β**_1–4_	1.0 (0%)				
Chronic bronchitis exacerbation	Number/year median, quartile 1–4 (n)	2.0; 1.5–3.0 (8)	3.0; 3.0–4.0 (17)	3.0; 2.0–3.0 (^#^ 6)	3.5; 1.1 (7)	n = 3	n = 3
		vs. Controls *p* =	0.2	0.3	**0.02**		
		**1-β**_1–4_	0.4 (60%)				
	Episode duration median, quartile 1–4 (n)	30.0; 15.0–45.0 (8)	21.0; 18.0–30.0 (17)	21.0; 18.8–24.0 (^#^ 6)	19.6; 5.1 (7)		
		vs. Controls *p* =	0.3	0.3	**0.02**		
		**1-β**_1–4_	1.0 (0%)				
Skin infection	Number/year median, quartile 1–4 (n)	1.0; 1.0–2.0 (8)	1.0; 1.0–6.0 (19)	1.0; 1.0–12.0 (11)	4.0; 4.0–6.0 (^#^ 5)	1.0; 1–12 (12)	n = 0
		vs. Controls *p* =	0.9	0.6	0.002	0.9	
		**1-β**_1–5_	0.2 (80%)				
	Episode duration median, quartile 1–4 (n)	8.5; 7.0–14.0 (8)	14.0; 10.0–20.0 (19)	14.0; 12.0–21.0(11)	10.0; 10.0–14.0 (^#^ 5)	13.0; 10–54 (12)	
		vs. Controls *p* =	0.3	0.1	0.9	0.4	
		**1-β**_1–5_	1.0 (0%)				

^ *p* ≤ 0.01—reliable difference (red); *p* ≥ 0.05—possible difference (hypothesis is notable and needs more study, blue). ^&^ aCCP neg/low—1–3 cut-off level, aCCP > 3 ULN—≥3× cut-off level. ^#^ The number of cases highlighted in red (n) in the cohort is too small (when the incidence of a particular infection is low) to obtain convincing results of statistical analysis. * 1-β—power of independence criteria in the aggregates of cohorts numbered in the column titles (Cramer’s V effect size). In parentheses—the probability (%) that as the sizes of the cohorts in an aggregate increase, a reliable difference in the indicators will appear or increase.

**Table 6:** Interconnection of annual infections and RA indices (Spearman rank order correlations).

Cohort	Indices	N	R	t(N-2)	*p*-Level	* 1-β
**All annual infection**						
eRAfam	Annual duration of all episodes and number of swollen joints (68 joint count)	48	0.31	2.24	0.03	0.4 (40%)
	Annual duration of all episodes and number of swollen joints (DAS28 joint count)	49	0.47	3.67	0.0007	0.9 (10%)
**Chronic tonsillitis exacerbation**						
eRAfam	Annual number of episodes and DAS28-ESR	13	−0.75	−3.8	0.003	0.8 (20%)
	Annual number of episodes and DAS28-CRP	12	−0.73	−3.4	0.007	0.7 (30%)
	Annual number of episodes and VAS	14	−0.55	−2.3	0.04	0.4 (60%)
	Annual number of episodes and HAQ	13	−0.69	−3.1	0.009	0.7 (30%)
	Annual duration of all episodes and DAS28-CRP	12	−0.58	−2.3	0.05	0.4 (60%)
	Annual duration of all episodes and VAS	14	−0.69	−3.3	0.006	0.7 (30%)
	Annual duration of all episodes and HAQ	13	−0.69	−3.2	0.009	0.7 (30%)
	Annual duration of all episodes and number of tender joints (68 joint count)	12	−0.63	−2.6	0.03	0.5 (50%)
^&^ eRA aCCP high	Annual duration of all episodes and number of tender joints (68 joint count)	12	−0.69	−3.0	0.01	0.6 (40%)
	Annual duration of all episodes and VAS	14	−0.68	−3.2	0.007	0.7 (30%)
	Annual duration of all episodes and HAQ	13	−0.57	−2.3	0.04	0.4 (60%)
**HSV exacerbations**						
^&^ eRA aCCP low/neg	One episode duration and DAS28-ESR	14	0.65	2.9	0.01	0.6 (40%)
	Annual duration of all episodes and CRP	13	0.83	4.9	0.0004	0.9 (10%)
	Annual number of episodes and CRP	13	0.77	3.9	0.002	0.8 (20%)
	One episode duration and CRP	13	0.81	4.5	0.0008	0.9 (10%)
**V-URI**						
eRAspo	One episode duration and ESR	34	0.35	2.1	0.04	0.4 (60%)
**URIab**						
eRAspo	Annual duration of all episodes and number of tender joints (68 joint count)	12	−0.77	−3.8	0.004	0.8 (20%)

^&^ aCCP neg/low—1–3 cut-off level, aCCP > 3 ULN—≥3x cut-off level. * 1-β—power of independence criteria (Cramer’s V effect size). In parentheses—the probability (%) that as the sizes of compared cohorts increase, a reliable difference in the indicators will appear or increase.

## Data Availability

The original contributions presented in the study are included in the article/supplementary material, further inquiries can be directed to the corresponding author/s.

## Supplementary Materials

The following supporting information can be downloaded at: https://www.mdpi.com/article/10.3390/jcm13102796/s1, Table S1: Probability of reliable correlation of RA indices and annual infection parameters in eRAall cohort with increasing sample size (1-β –power of independence criteria, Cramer’s V effect size); Figure S1: Publications on the problem of the trigger role of infections in rheumatoid arthritis development: A: All publications. B: Reviews.

## References

[1] Arleevskaya M., Takha E., Petrov S., Kazarian G., Novikov A., Larionova R., Valeeva A., Shuralev E., Mukminov M., Bost C. (2021). Causal risk and protective factors in rheumatoid arthritis: A genetic update. J. Transl. Autoimmun..

[2] Arleevskaya M.I., Aminov R., Brooks W.H., Manukyan G., Renaudineau Y. (2019). Editorial: Shaping of Human Immune System and Metabolic Processes by Viruses and Microorganisms. Front. Microbiol..

[3] Arleevskaya M.I., Kravtsova O.A., Lemerle J., Renaudineau Y., Tsibulkin A.P. (2016). How Rheumatoid Arthritis Can Result from Provocation of the Immune System by Microorganisms and Viruses. Front. Microbiol..

[4] Arleevskaya M.I., Gabdoulkhakova A.G., Filina Y.V., Miftakhova R.R., Bredberg A., Tsybulkin A.P. (2014). A transient peak of infections during onset of rheumatoid arthritis: A 10-year prospective cohort study. BMJ Open.

[5] Larionova R.V., Arleevskaya M.I., Kravtsova O.A., Validov S., Renaudineau Y. (2019). In seroconverted rheumatoid arthritis patients a multi-reactive anti-herpes IgM profile is associated with disease activity. Clin. Immunol..

[6] Jonsson H., Helgason J. (1996). Rheumatoid Arthritis in an Icelandic Textbook from 1782. Scand. J. Rheumatol..

[7] Weyand C.M., Klimiuk P.A., Goronzy J.J. (1998). Heterogeneity of rheumatoid arthritis: From phenotypes to genotypes. Springer Seminars in Immunopathology.

[8] Arleevskaya M.I., Boulygina E.A., Larionova R., Validov S., Kravtsova O., Shagimardanova E.I., Velo L., Hery-Arnaud G., Carlé C., Renaudineau Y. (2022). Anti-Citrullinated Peptide Antibodies Control Oral *Porphyromonas* and *Aggregatibacter species* in Patients with Rheumatoid Arthritis. Int. J. Mol. Sci..

[9] Aletaha D., Neogi T., Silman A.J., Funovits J., Felson D.T., Bingham C.O., Birnbaum N.S., Burmester G.R., Bykerk V.P., Cohen M.D. (2010). 2010 Rheumatoid arthritis classification criteria: An American College of Rheumatology/European League Against Rheumatism collaborative initiative. Ann. Rheum. Dis..

[10] Arleevskaya M.I., Larionova R.V., Shagimardanova E.I., Gogoleva N.E., Kravtsova O.A., Novikov A.A., Kazarian G.G., Carlé C., Renaudineau Y. (2023). Predictive risk factors before the onset of familial rheumatoid arthritis: The Tatarstan cohort study. Front. Med..

[11] Söderlin M.K., Bergsten U., Svensson B., BARFOT Study Group (2010). Patient-reported events preceding the onset of rheumatoid arthritis: Possible clues to aetiology. Musculoskelet. Care.

[12] Arleevskaya M.I., Shafigullina A.Z., Filina Y.V., Lemerle J., Renaudineau Y. (2017). Associations between Viral Infection History Symptoms, Granulocyte Reactive Oxygen Species Activity, and Active Rheumatoid Arthritis Disease in Untreated Women at Onset: Results from a Longitudinal Cohort Study of Tatarstan Women. Front. Immunol..

[13] Champely S., Ekstrom C., Dalgaard P., Gill J., Weibelzahl S., Anandkumar A., Ford C., Volcic R., De Rosario H. pwr: Basic Functions for Power Analysis CRAN Date/Publication—2020-03-17 12:10:02 UTC. https://github.com/heliosdrm/pwr.

[14] Cohen J. (1988). Statistical Power Analysis for the Behavioral Sciences.

[15] Kolassa J.E., Jankowski S. MultNonParam: Multivariate Nonparametric Methods. https://CRAN.R-project.org/package=MultNonParam.

[16] Augusto D.G., Murdolo L.D., Chatzileontiadou D.S., Sabatino J.J., Yusufali T., Peyser N.D., Butcher X., Kizer K., Guthrie K., Murray V.W. (2023). A common allele of HLA is associated with asymptomatic SARS-CoV-2 infection. Nature.

[17] Kesson A.M. (2001). Management of Neonatal Herpes Simplex Virus Infection. Pediatr. Drugs.

[18] Tanaka N. (2001). Infection of herpes simplex virus (HSV) and Epstein-Barr virus (EBV) in acute tonsillitis–histopathological as-sessment by optical and electron microscopic observation of biopsy specimens of tonsils. Nihon Jibiinkoka Gakkai Kaiho.

[19] Rusan M., Klug T.E., Henriksen J.J., Ellermann-Eriksen S., Fuursted K., Ovesen T. (2012). The role of viruses in the pathogenesis of peritonsillar abscess. Eur. J. Clin. Microbiol. Infect. Dis..

[20] Chayavichitsilp P., Buckwalter J.V., Krakowski A.C., Friedlander S.F. (2009). Herpes simplex. Pediatr Rev..

[21] Grinde B. (2013). Herpesviruses: Latency and reactivation–viral strategies and host response. J. Oral Microbiol..

[22] Akhtar J., Shukla D. (2009). Viral entry mechanisms: Cellular and viral mediators of herpes simplex virus entry. FEBS J..

[23] Bottini N., Firestein G.S. (2013). Epigenetics in Rheumatoid Arthritis: A Primer for Rheumatologists. Curr. Rheumatol. Rep..

[24] Kang Y.M., Kim S.Y., Kang J.H., Han S.W., Nam E.J., Kyung H.S., Park J.Y., Kim I.S. (2007). Light up-regulated on B lymphocytes and monocytes in rheumatoid arthritis mediates cellular adhesion and metalloproteinase production by synoviocytes. Arthritis Rheum..

[25] Shang Y., Guo G., Cui Q., Li J., Ruan Z., Chen Y. (2011). The Expression and Anatomical Distribution of BTLA and Its Ligand HVEM in Rheumatoid Synovium. Inflammation.

[26] Jung H.W., La S.J., Kim J.Y., Heo S.K., Kim J.Y., Wang S., Kim K.K., Lee K.M., Cho H.R., Lee H.W. (2003). High levels of soluble herpes virus entry mediator in sera of patients with allergic and autoimmune diseases. Exp. Mol. Med..

[27] Nakano K., Asano R., Tsumoto K., Kwon H., Goins W.F., Kumagai I., Cohen J.B., Glorioso J.C. (2005). Herpes simplex virus targeting to the EGF receptor by a gD-specific soluble bridging molecule. Mol Ther..

[28] Nakamura N., Shimaoka Y., Tougan T., Onda H., Okuzaki D., Zhao H., Fujimori A., Yabuta N., Nagamori I., Tanigawa A. (2006). Isolation and Expression Profiling of Genes Upregulated in Bone Marrow-Derived Mononuclear Cells of Rheumatoid Arthritis Patients. DNA Res..

[29] Lo S.-F., Wan L., Lin H.-C., Huang C.-M., Chen S.-Y., Liu S.-C., Tsai F.-J. (2011). Association of rheumatoid arthritis risk with EGFR genetic polymorphisms in Taiwan’s Han Chinese population. Rheumatol. Int..

[30] Yuan F.L., Li X., Lu W.G., Sun J.M., Jiang D.L., Xu R.S. (2013). Epidermal growth factor receptor (EGFR) as a therapeutic target in rheu-matoid arthritis. Clin. Rheumatol..

[31] Summerfield J.A., Ryder S., Sumiya M., Thursz M., Gorchein A., Monteil M.A., Turner M.W. (1995). Mannose binding protein gene mutations associated with unusual and severe infections in adults. Lancet.

[32] Seppänen M., Lokki M.-L., Lappalainen M., Hiltunen-Back E., Rovio A.T., Kares S., Hurme M., Aittoniemi J. (2009). Mannose-binding lectin 2 gene polymorphism in recurrent herpes simplex virus 2 infection. Hum. Immunol..

[33] Put S., Schoonooghe S., Devoogdt N., Schurgers E., Avau A., Mitera T., D’huyvetter M., De Baetselier P., Raes G., Lahoutte T. (2013). SPECT Imaging of Joint Inflammation with Nanobodies Targeting the Macrophage Mannose Receptor in a Mouse Model for Rheumatoid Arthritis. J. Nucl. Med..

[34] Jacobsen S., Madsen H.O., Klarlund M.E.T.T.E., Jensen T.R.I.N.E., Skjødt H.E.N.R.I.K., Jensen K.E., Svejgaard A., Garred P., TIRA Group (2001). The influence of mannose binding lectin polymorphisms on disease outcome in early polyarthritis. J. Rheumatol..

[35] Saevarsdottir S., Vikingsdottir T., Vikingsson A., Manfredsdottir V., Geirsson A.J., Valdimarsson H. (2001). Low mannose binding lectin predicts poor prognosis in patients with early rheumatoid arthritis. A prospective study. J. Rheumatol..

[36] Olsen N., Sokka T., Seehorn C.L., Kraft B., Maas K., Moore J., Aune T.M. (2004). A gene expression signature for recent onset rheumatoid arthritis in peripheral blood mononuclear cells. Ann. Rheum. Dis..

[37] Ip W.K., Lau Y.L., Chan S.Y., Mok C.C., Chan D., Tong K.K., Lau C.S. (2000). Mannose-binding lectin and rheumatoid arthritis in southern Chinese. Arthritis Rheum..

[38] Khanna K.M., Bonneau R.H., Kinchington P.R., Hendricks R.L. (2003). Herpes simplex virus-specific memory CD8+ T cells are selectively activated and retained in latently infected sensory ganglia. Immunity.

[39] Khanna K.M., Lepisto A.J., Decman V., Hendricks R.L. (2004). Immune control of herpes simplex virus during latency. Curr. Opin. Immunol..

[40] Egan K.P., Wu S., Wigdahl B., Jennings S.R. (2013). Immunological control of herpes simplex virus infections. J. NeuroVirology.

[41] Minami M., Kita M., Yan X.Q., Yamamoto T., Iida T., Sekikawa K., Iwakura Y., Imanishi J. (2002). Role of IFN-gamma and tumor necrosis factor-alpha in herpes simplex virus type 1 infection. J. Interferon Cytokine Res..

[42] Davis J.M., Knutson K.L., Strausbauch M.A., Green A.B., Crowson C.S., Therneau T.M., Matteson E.L., Gabriel S.E. (2013). Immune response profiling in early rheumatoid arthritis: Discovery of a novel interaction of treatment response with viral immunity. Arthritis Res. Ther..

[43] Motamedifar M., Sarvari J., Ebrahimpour A., Emami A. (2015). Symptomatic Reactivation of HSV Infection Correlates with Decreased Serum Levels of TNF-α. Iran J. Immunol..

[44] Moss D.J., Klestov A., Burrows S., Kane R.G. (1983). A comparison of Epstein-Barr virus-specific T-cell immunity in rheumatoid arthritis and osteoarthritis patients. Aust. J. Exp. Biol. Med. Sci..

[45] Gaston J.S., Rickinson A.B., Yao Q.Y., Epstein M.A. (1986). The abnormal cytotoxic T cell response to Epstein-Barr virus in rheumatoid arthritis is correlated with disease activity and occurs in other arthropathies. Ann. Rheum. Dis..

[46] Toussirot E., Wendling D., Tiberghien P., Luka J., Roudier J. (2000). Decreased T cell precursor frequencies to Epstein-Barr virus glycoprotein Gp110 in peripheral blood correlate with disease activity and severity in patients with rheumatoid arthritis. Ann. Rheum. Dis..

[47] Ahmed-Hassan H., Sisson B., Shukla R.K., Wijewantha Y., Funderburg N.T., Li Z., Hayes D., Demberg T., Liyanage N.P.M. (2020). Innate Immune Responses to Highly Pathogenic Coronaviruses and Other Significant Respiratory Viral Infections. Front. Immunol..

[48] McCullers J.A. (2014). The co-pathogenesis of influenza viruses with bacteria in the lung. Nat. Rev. Microbiol..

[49] Prasso J.E., Deng J.C. (2017). Postviral Complications: Bacterial Pneumonia. Clin. Chest Med..

[50] Mina M.J., McCullers J.A., Klugman K.P. (2014). Live attenuated influenza vaccine enhances colonization of Streptococcus pneumoniae and Staphylococcus aureus in mice. mBio.

[51] Hanada S., Pirzadeh M., Carver K.Y., Deng J.C. (2018). Respiratory Viral Infection-Induced Microbiome Alterations and Secondary Bacterial Pneumonia. Front. Immunol..

[52] Kudva A., Scheller E.V., Robinson K.M., Crowe C.R., Choi S.M., Slight S.R., Khader S.A., Dubin P.J., Enelow R.I., Kolls J.K. (2011). Influenza A inhibits Th17-mediated host defense against bacterial pneumonia in mice. J Immunol..

[53] Castañeda-Delgado J.E., Bastián-Hernandez Y., Macias-Segura N., Santiago-Algarra D., Castillo-Ortiz J.D., Alemán-Navarro A.L., Martínez-Tejada P., Enciso-Moreno L., Garcia-De Lira Y., Olguín-Calderón D. (2017). Type I Interferon Gene Response Is Increased in Early and Established Rheumatoid Arthritis and Correlates with Autoantibody Production. Front Immunol..

[54] Gomez J.C., Yamada M., Martin J.R., Dang H., Brickey W.J., Bergmeier W., Dinauer M.C., Doerschuk C.M. (2015). Mechanisms of interferon-γ production by neutrophils and its function during Streptococcus pneumoniae pneumonia. Am. J. Respir. Cell Mol. Biol..

[55] Arleevskaya M.I., Gabdoulkhakova A.G., Filina J.V., Zabotin A.I., Tsibulkin A.P. (2011). Mononuclear Phagocytes in Rheumatoid Arthritis Patients and their Relatives-Family Similarity. Open Rheumatol. J..

[56] Olsson L.M., Lindqvist A.-K., Källberg H., Padyukov L., Burkhardt H., Alfredsson L., Klareskog L., Holmdahl R. (2007). A case-control study of rheumatoid arthritis identifies an associated single nucleotide polymorphism in the NCF4 gene, supporting a role for the NADPH-oxidase complex in autoimmunity. Arthritis Res. Ther..

[57] Vuononvirta J., Toivonen L., Gröndahl-Yli-Hannuksela K., Barkoff A.M., Lindholm L., Mertsola J., Peltola V., He Q. (2011). Nasopha-ryngeal bacterial colonization and gene polymorphisms of mannose-binding lectin and toll-like receptors 2 and 4 in infants. PLoS ONE.

[58] Edwards C.J., Feldman J.L., Beech J., Shields K.M., Stover J.A., Trepicchio W.L., Larsen G., Foxwell B.M.J., Brennan F.M., Feldmann M. (2007). Molecular Profile of Peripheral Blood Mononuclear Cells from Patients with Rheumatoid Arthritis. Mol. Med..

[59] Cole A.L., Muthukrishnan G., Chong C., Beavis A., Eade C.R., Wood M.P., Deichen M.G., Cole A.M. (2016). Host innate inflammatory factors and staphylococcal protein A influence the duration of human Staphylococcus aureus nasal carriage. Mucosal Immunol..

[60] DeLorenze G.N., Nelson C.L., Scott W.K., Allen A.S., Ray G.T., Tsai A.-L., Quesenberry C.P., Fowler V.G. (2015). Polymorphisms in HLA Class II Genes Are Associated With Susceptibility to *Staphylococcus aureus* Infection in a White Population. J. Infect. Dis..

[61] Dieperink S.S., Glintborg B., Oestergaard L.B., Nørgaard M., Benfield T., Mehnert F., Petersen A., Hetland M.L. (2019). Risk factors for Staphylococcus aureus bacteremia in patients with rheumatoid arthritis and incidence compared with the general population: Protocol for a Danish nationwide observational cohort study. BMJ Open.

[62] Ataee R.A., Kashefi R., Alishiri G.H., Esmaieli D. (2015). Assay of Blood and Synovial Fluid of Patients with Rheumatoid Arthritis for Staphylococcus aureus Enterotoxin D: Absence of Bacteria But Presence of Its Toxin. Jundishapur J. Microbiol..

[63] Utz P.J., Genovese M.C., Robinson W.H. (2004). Unlocking the “PAD” lock on rheumatoid arthritis. Ann. Rheum. Dis..

[64] Kim V., Criner G.J. (2013). Chronic Bronchitis and Chronic Obstructive Pulmonary Disease. Am. J. Respir. Crit. Care Med..

[65] Saltanova Z.E. (2015). Chronic tonsillitis, etiological and pathogenetic aspects of the development of metatonsillar complications. Vestn Otorinolaringol..

[66] Bondareva G.P., Antonova N.A., Chumakov P.L. (2013). Immunomorphological features of chronic tonsillitis. Vestn Otorinolaringol..

[67] Klug T.E., Henriksen J.-J., Rusan M., Fuursted K., Krogfelt K.A., Ovesen T., Struve C. (2014). Antibody development to *Fusobacterium necrophorum* in patients with peritonsillar abscess. Eur. J. Clin. Microbiol. Infect. Dis..

[68] Ahearn C.P., Gallo M.C., Murphy T.F. (2017). Insights on persistent airway infection by non-typeable Haemophilus influenzae in chronic obstructive pulmonary disease. Pathog. Dis..

[69] Wang B., Dileepan T., Briscoe S., Hyland K.A., Kang J., Khoruts A., Cleary P.P. (2010). Induction of TGF-beta1 and TGF-beta1-dependent predominant Th17 differentiation by group A streptococcal infection. Proc. Natl. Acad. Sci. USA.

[70] Wang S., Wang S., Li H., Zhu L., Wang Y. (2019). Inhibition of the TGF-β/Smads signaling pathway attenuates pulmonary fibrosis and induces anti-proliferative effect on synovial fibroblasts in rheumatoid arthritis. Int. J. Clin. Exp. Pathol..

[71] Lee Y.H., Bae S.C. (2017). Association between circulating transforming growth factor-β1 level and polymorphisms in systemic lupus erythematosus and rheumatoid arthritis: A meta-analysis. Cell Mol. Biol..

[72] Zhu D., Zhao J., Lou A., Huang Q., OuYang Q., Zhu J., Fan M., He Y., Ren H., Yang M. (2019). Transforming growth factor β1 promotes fibroblast-like synoviocytes migration and invasion via TGF-β1/Smad signaling in rheumatoid arthritis. Mol. Cell Biochem..

[73] Gideon H.P., Flynn J.L. (2011). Latent tuberculosis: What the host “sees”?. Immunol. Res..

[74] Carmona L., Hernández-García C., Vadillo C., Pato E., Balsa A., González-Alvaro I., Belmonte M.A., Tena X., Sanmartí R., EMECAR Study Group (2003). Increased risk of tuberculosis in patients with rheumatoid arthritis. J. Rheumatol..

[75] Bekale R.B., Du Plessis S.M., Hsu N.J., Sharma J.R., Sampson S.L., Jacobs M., Meyer M., Morse G.D., Dube A. (2018). Mycobacterium Tuberculosis and Interactions with the Host Immune System: Opportunities for Nanoparticle Based Immunotherapeutics and Vaccines. Pharm Res..

[76] Sia J.K., Georgieva M., Rengarajan J. (2015). Innate Immune Defenses in Human Tuberculosis: An Overview of the Interactions between Mycobacterium tuberculosis and Innate Immune Cells. J Immunol Res..

[77] Gupta N., Kumar R., Agrawal B. (2018). New Players in Immunity to Tuberculosis: The Host Microbiome, Lung Epithelium, and Innate Immune Cells. Front. Immunol..

[78] Perry S., Chang A.H., Sanchez L., Yang S., Haggerty T.D., Parsonnet J. (2013). The immune response to tuberculosis infection in the setting of Helicobacter pylori and helminth infections. Epidemiol. Infect..

[79] Segal L.N., Clemente J.C., Li Y., Ruan C., Cao J., Danckers M., Morris A., Tapyrik S., Wu B.G., Diaz P. (2017). Anaerobic Bacterial Fermentation Products Increase Tuberculosis Risk in Antiretroviral-Drug-Treated HIV Patients. Cell Host Microbe.

[80] Aravindan P.P. (2019). Host genetics and tuberculosis: Theory of genetic polymorphism and tuberculosis. Lung India.

[81] Fernández-Mestre M., Villasmil Á., Takiff H., Fuentes Alcala Z. (2015). NRAMP1 and VDR Gene Polymorphisms in Susceptibility to Tuberculosis in Venezuelan Population. Dis. Markers.

[82] Correa M.A., Canhamero T., Borrego A., Katz I.S., Jensen J.R., Guerra J.L., Cabrera W.H., Starobinas N., Fernandes J.G., Ribeiro O.G. (2017). *Slc11a1* (*Nramp*-1) gene modulates immune-inflammation genes in macrophages during pristane-induced arthritis in mice. Inflamm. Res..

[83] Pai M., Behr M.A., Dowdy D., Dheda K., Divangahi M., Boehme C.C., Ginsberg A., Swaminathan S., Spigelman M., Getahun H. (2016). Tuberculosis. Nat. Rev. Dis. Primers.

[84] Satti I., McShane H. (2018). Current approaches toward identifying a correlate of immune protection from tuberculosis. Expert Rev. Vaccines.

[85] Namasivayam S., Sher A., Glickman M.S., Wipperman M.F. (2018). The Microbiome and Tuberculosis: Early Evidence for Cross Talk. mBio.

[86] Nash A.A. (2000). T Cells and the Regulation of Herpes Simplex Virus Latency and Reactivation. J. Exp. Med..

[87] Borysiewicz L.K., Graham S., Hickling J.K., Mason P.D., Sissons J.G.P. (1988). Human cytomegalovirus-specific cytotoxic T cells: Their precursor frequency and stage specificity. Eur. J. Immunol..

[88] Posavad C.M., Huang M.L., Barcy S., Koelle D.M., Corey L. (2000). Long Term Persistence of Herpes Simplex Virus-Specific CD8+ CTL in Persons with Frequently Recurring Genital Herpes. J. Immunol..

[89] van Lint A.L., Kleinert L., Clarke S.R., Stock A., Heath W.R., Carbone F.R. (2005). Latent infection with herpes simplex virus is associated with ongoing CD8+ T-cell stimulation by parenchymal cells within sensory ganglia. J Virol..

[90] Deshpande S.P., Kumaraguru U., Rouse B.T. (2000). Dual Role of B Cells in Mediating Innate and Acquired Immunity to Herpes Simplex Virus Infections. Cell. Immunol..

[91] Youinou P., Jamin C., PERS J.O., Berthou C., Saraux A., Renaudineau Y. (2005). B lymphocytes are required for development and treatment of autoimmune diseases. Ann. N. Y. Acad. Sci..

[92] Gazeau P., Devauchelle-Pensec V., Pochard P., Pers J.-O., Saraux A., Renaudineau Y., Cornec D. (2015). Abatacept efficacy in rheumatoid arthritis is dependent upon baseline blood B-cell levels. Rheumatology.

[93] Heo S.-K., Ju S.-A., Lee S.-C., Park S.-M., Choe S.-Y., Kwon B., Kwon B.S., Kim B.-S. (2005). LIGHT enhances the bactericidal activity of human monocytes and neutrophils via HVEM. J. Leukoc. Biol..

[94] Arleevskaya M.I., Larionova R.V., Brooks W.H., Bettacchioli E., Renaudineau Y. (2020). Toll-Like Receptors, Infections, and Rheumatoid Arthritis. Clin. Rev. Allergy Immunol..

[95] Plenge R.M. (2009). Recent progress in rheumatoid arthritis genetics: One step towards improved patient care. Curr. Opin. Rheumatol..

[96] Oki M., Watanabe N., Owada T., Oya Y., Ikeda K., Saito Y., Matsumura R., Seto Y., Iwamoto I., Nakajima H. (2011). A functional poly-morphism in B and T lymphocyte attenuator is associated with susceptibility to rheumatoid arthritis. Clin. Dev. Immunol..

[97] Kahmini F.R., Shahgaldi S., Azimi M., Mansourabadi A.H. (2022). Emerging therapeutic potential of regulatory T (Treg) cells for rheumatoid arthritis: New insights and challenges. Int. Immunopharmacol..

[98] Pérol L., Lindner J.M., Caudana P., Nunez N.G., Baeyens A., Valle A., Sedlik C., Loirat D., Boyer O., Créange A. (2016). Loss of immune tolerance to IL-2 in type 1 diabetes. Nat. Commun..

[99] Bo M., Niegowska M., Eames H.L., Almuttaqi H., Arru G., Erre G.L., Passiu G., Khoyratty T.E., van Grinsven E., Udalova I.A. (2020). Antibody response to homologous epitopes of Epstein-Barr virus, Mycobacterium avium subsp. paratuberculosis and IRF5 in patients with different connective tissue diseases and in mouse model of antigen-induced arthritis. J. Transl. Autoimmun..

[100] Mei X.X., Lei S.S., Xu L., Wu S., Gu H.P., Du Y., Zhao T., Xie G.Q., Fan Y.S., Pan X.P. (2020). Herpes simplex virus type I-infected disorders alter the balance between Treg and Th17 cells in recurrent herpes labialis patients. Int. J. Immunopathol. Pharmacol..

[101] Parton C., Ussher J.M., Perz J. (2022). Mothers’ experiences of wellbeing and coping while living with rheumatoid arthritis: A qual-itative study. BMC Womens Health.

[102] Al Juffali L., Almalag H.M., Alswyan N., Almutairi J., Alsanea D., Alarfaj H.F., Alarfaj A.S., Abouzaid H.H., Omair M.A. (2022). The Patient Activation Measure in Patients with Rheumatoid Arthritis: A Systematic Review and Cross-Sectional Interview-Based Survey. Patient Prefer. Adherence.

[103] Almalag H.M., Almuhareb A.M., Alsharafi A.A., Alhawassi T.M., Alghamdi A.A., Alarfaj H., Omair M.A., Alomari B.A., Alblowi M.S., Abouzaid H.H. (2021). Relationship between different anti-rheumatic drug therapies and complementary and alternative medicine in patients with rheumatoid arthritis: An interview based cross-sectional study. Saudi Pharm. J..

[104] Wæhrens E.E., Bliddal H., Danneskiold-Samsøe B., Lund H., Fisher A.G. (2012). Differences between questionnaire- and interview-based measures of activities of daily living (ADL) ability and their association with observed ADL ability in women with rheumatoid arthritis, knee osteoarthritis, and fibromyalgia. Scand. J. Rheumatol..

[105] Björk M., Thyberg I., Valtersson E., Östlund G., Stenström B., Sverker A. (2018). Foot Barriers in Patients With Early Rheumatoid Arthritis: An Interview Study Among Swedish Women and Men. Arthritis Care Res..

