# Supplementary material for: At Early Rheumatoid Arthritis Stage, the Infectious Spectrum Is Driven by Non-Familial Factors and Anti-CCP Immunization
Source: J Clin Med. 2024 May 9;13(10):2796. doi: 10.3390/jcm13102796 (PMC11122272; doi:10.3390/jcm13102796)
Supplement: Supplementary file 1 [file jcm-13-02796-s001.zip › jcm-2951715-supplementary.pdf]

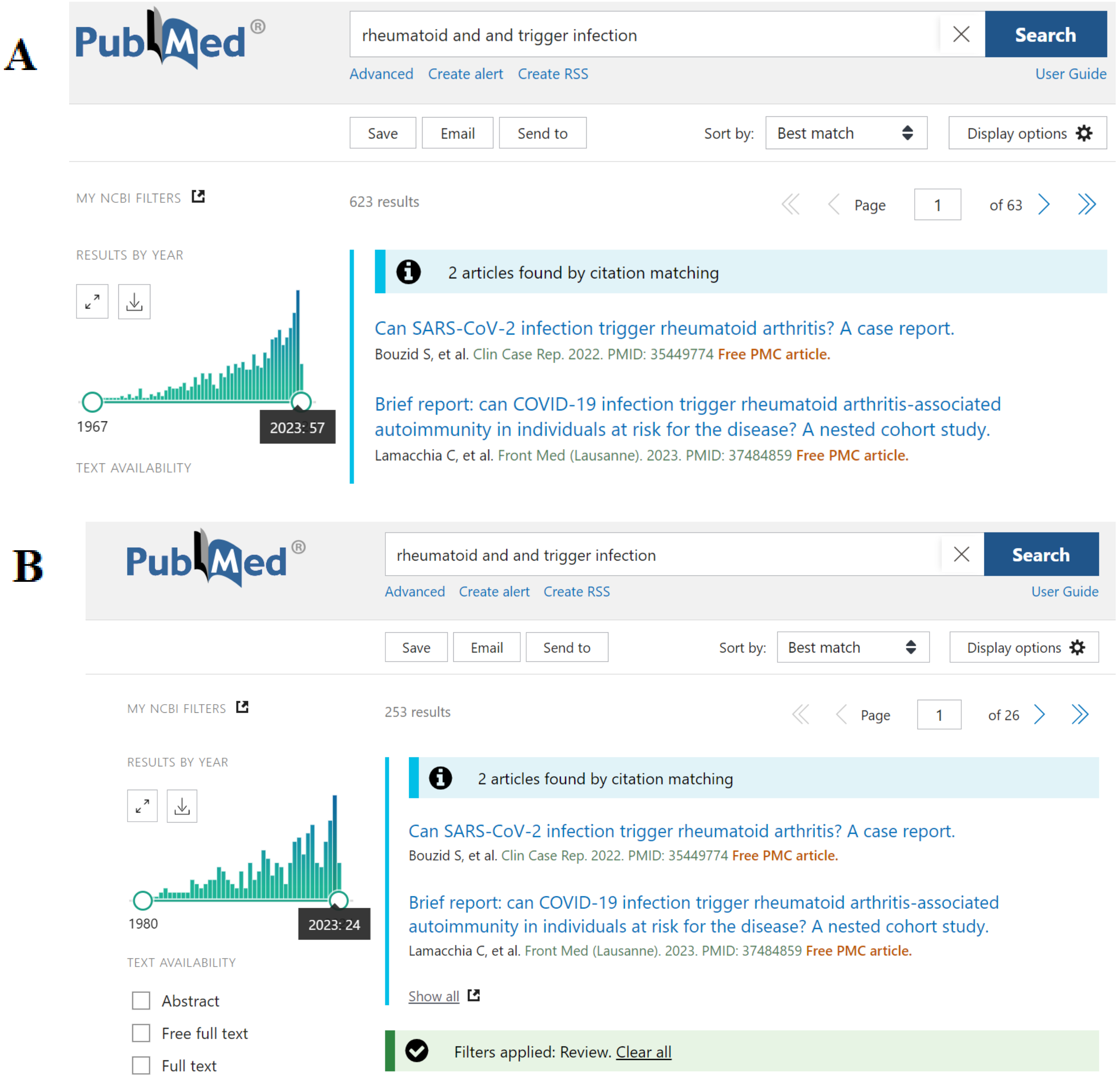

Supplementary Figure S1 Publications on the problem of the trigger role of infections in rheumatoid arthritis development: A: All publications. B: Reviews.

Supplementary Table S1. Probability of reliable correlation of RA indices and annual infection parameters in eRAall cohort with increasing sample size (1-β –power of independence criteria, Cramer's V effect size)

Analized indices	N	Spearman	t.N.2.	p.value	1-beta	Probability of reliable correlation with increasing sample size, %
**All infection burden**						
Serum aCCP level & Annual duration of all episodes	115	-0.172276	-1.85911	0.065613	0,46	54,44
Serum aCCP leve & One episode duration	115	-0.216362	-2.35576	0.020208	0,65	35,37
Serum aCCP level & Annual number of episodes	115	-0.049987	-0.53204	0.595746	0,08	**91,72**
CRP & Annual duration of all episodes	144	-0.02322	-0.27678	0.782355	0,06	**94,13**
CRP & One episode duration	144	-0.0705	-0.8422	0.401095	0,13	**86,60**
CRP & Annual number of episodes	144	0.045482	0.54254	0.588299	0,08	**91,58**
DASCRP & Annual duration of all episodes	76	0.10952	0.94783	0.3463	0,16	**84,34**
DASCRP & One episode duration	76	-0.005559	-0.04782	0.961989	0,05	**94,99**
DASCRP & Annual number of episodes	76	0.177309	1.54982	0.125451	0,34	**66,27**
DASESR & Annual duration of all episodes	117	0.09137	0.98395	0.327207	0,17	**83,45**
DASESR & One episode duration	117	0.020329	0.21805	0.827774	0,06	**94,47**
DASESR & Annual number of episodes	117	0.144897	1.57042	0.119066	0,35	**65,39**
ESR & Annual duration of all episodes	154	0.045313	0.55923	0.576826	0,09	**91,36**
ESR & One episode duration	154	0.075929	0.93883	0.34931	0,16	**84,50**
ESR & Annual number of episodes	154	-0.077631	-0.95999	0.338584	0,16	**84,00**
HAQ & Annual duration of all episodes	102	-0.228094	-2.3427	0.021126	0,64	35,94
HAQ & One episode duration	102	-0.200031	-2.04157	0.043827	0,53	47,36
HAQ & Annual number of episodes	102	-0.080555	-0.80818	0.420905	0,13	**87,30**
Morning stiffness, hours & Annual duration of all episodes	138	0.083912	0.98203	0.327827	0,17	**83,49**
Morning stiffness, hours & One episode duration	138	0.11133	1.30644	0.193607	0,26	**74,40**
Morning stiffness, hours & Annual number of episodes	138	0.043843	0.51179	0.609631	0,08	**91,97**
Serum RF level & Annual duration of all episodes	137	0.113963	1.33282	0.184837	0,26	**73,55**

Serum RF level & One episode duration	137	0.123176	1.44216	0.151573	0,30	**69,90**
Serum RF level & Annual number of episodes	137	0.009503	0.11042	0.912242	0,05	**94,87**
Number of swollen joints (DAS28 joint count) & Annual duration of all episodes	85	0.312717	2.99942	0.00357	0,84	16,23
Number of swollen joints (DAS28 joint count) & One episode duration	85	0.276851	2.62483	0.010318	0,73	26,52
Number of swollen joints (DAS28 joint count) & Annual number of episodes	85	0.150027	1.38246	0.17054	0,28	**72,02**
Number of swollen joints (68 joint count) & Annual duration of all episodes	84	0.22592	2.10008	0.038795	0,55	45,24
Number of swollen joints (68 joint count) & One episode duration	84	0.189089	1.74373	0.084954	0,41	**59,04**
Number of swollen joints (68 joint count) & Annual number of episodes	84	0.13868	1.26806	0.208366	0,24	**75,67**
Number of tender joints (28 joint count) & Annual duration of all episodes	85	0.045979	0.41933	0.676058	0,07	**92,99**
Number of tender joints (28 joint count) & One episode duration	85	0.029786	0.27148	0.786696	0,06	**94,17**
Number of tender joints (28 joint count) & Annual number of episodes	85	0.036138	0.32945	0.742649	0,06	**93,77**
Number of tender joints (68 joint count) & Annual duration of all episodes	85	-0.156555	-1.44409	0.152479	0,30	**69,94**
Number of tender joints (68 joint count) & One episode duration	85	-0.122672	-1.1261	0.263368	0,20	**79,81**
Number of tender joints (68 joint count) & Annual number of episodes	85	-0.058287	-0.53193	0.596197	0,08	**91,73**
VAS & Annual duration of all episodes	127	-0.044517	-0.49821	0.619209	0,08	**92,13**
VAS & One episode duration	127	-0.070282	-0.78772	0.432351	0,12	**87,68**
VAS & Annual number of episodes	127	0.020007	0.22373	0.823334	0,06	**94,44**
**Acute otitis**						
Serum aCCP level & Annual duration of all episodes	17	-0.007521	-0.02913	0.977146	0,05	**95,00**
Serum aCCP level & One episode duration	17	0.166389	0.65353	0.523305	0,10	**90,20**
Serum aCCP level & Annual number of episodes	17	-0.14859	-0.58195	0.569245	0,09	**91,22**
CRP & Annual duration of all episodes	24	0.145966	0.69205	0.496144	0,10	**89,52**
CRP & One episode duration	24	-0.052237	-0.24535	0.808463	0,06	**94,38**
CRP &Annual number of episodes	24	0.114894	0.5425	0.592931	0,08	**91,68**
DASCRP & Annual duration of all episodes	11	0.344279	1.10009	0.299846	0,18	**81,63**
DASCRP & One episode duration	11	-0.140921	-0.42702	0.679395	0,07	**93,08**
DASCRP & Annual number of episodes	11	0.205509	0.62997	0.544372	0,09	**90,68**

DASESR & Annual duration of all episodes	20	0.3116	1.39128	0.1811	0,27	72,65
DASESR & One episode duration	20	-0.28193	-1.2467	0.228487	0,23	76,99
DASESR & Annual number of episodes	20	0.401632	1.86064	0.079212	0,43	56,66
ESR & Annual duration of all episodes	25	-0.307277	-1.54857	0.135137	0,33	67,19
ESR & One episode duration	25	0.189178	0.92395	0.365105	0,15	85,08
ESR & Annual number of episodes	25	-0.348425	-1.7827	0.087841	0,41	58,91
HAQ & Annual duration of all episodes	17	0.495946	2.21199	0.042901	0,55	44,99
HAQ & One episode duration	17	0.043511	0.16868	0.868305	0,05	94,76
HAQ & Annual number of episodes	17	0.374749	1.56548	0.13832	0,33	67,32
Morning stiffness, hours & Annual duration of all episodes	23	0.196211	0.91697	0.369567	0,15	85,26
Morning stiffness, hours & One episode duration	23	0.224386	1.05517	0.303335	0,18	82,04
Morning stiffness, hours & Annual number of episodes	23	0.02601	0.11923	0.906225	0,05	94,90
Serum RF level & Annual duration of all episodes	22	0.187731	0.85475	0.40281	0,13	86,58
Serum RF level & One episode duration	22	0.075884	0.34035	0.737145	0,06	93,75
Serum RF level & Annual number of episodes	22	0.066282	0.29707	0.769474	0,06	94,07
Number of swollen joints (DAS28 joint count) & Annual duration of all episodes	16	0.046003	0.17231	0.865659	0,05	94,76
Number of swollen joints (DAS28 joint count) & One episode duration	16	0.076456	0.28691	0.778383	0,06	94,16
Number of swollen joints (DAS28 joint count) & Annual number of episodes	16	-0.061645	-0.23109	0.820586	0,06	94,49
Number of swollen joints (68 joint count) & Annual duration of all episodes	16	0.121077	0.45639	0.655111	0,07	92,73
Number of swollen joints (68 joint count) & One episode duration	16	0.189425	0.72183	0.48228	0,11	89,13
Number of swollen joints (68 joint count) & Annual number of episodes	16	-0.07458	-0.27983	0.7837	0,06	94,21
Number of tender joints (28 joint count) & Annual duration of all episodes	16	0.07199	0.27006	0.791053	0,06	94,27
Number of tender joints (28 joint count) & One episode duration	16	0.326093	1.29068	0.217723	0,24	75,99
Number of tender joints (28 joint count) & Annual number of episodes	16	-0.165037	-0.6261	0.541324	0,09	90,62
Number of tender joints (68 joint count) & Annual duration of all episodes	16	0.130696	0.49325	0.629482	0,08	92,33
Number of tender joints (68 joint count) & One episode duration	16	0.438383	1.82499	0.08941	0,41	58,68
Number of tender joints (68 joint count) & Annual number of episodes	16	-0.107029	-0.40278	0.693193	0,07	93,25

VAS & Annual duration of all episodes	22	0.221433	1.01549	0.322001	0,17	**83,03**
VAS & One episode duration	22	-0.160669	-0.72799	0.475056	0,11	**88,94**
VAS & Annual number of episodes	22	0.267896	1.24352	0.228056	0,23	**77,00**
**Chronic otitis exacerbations**						
Serum aCCP level & Annual duration of all episodes	18	0.002587	0.01035	0.991873	0,05	**95,08**
Serum aCCP level & One episode duration	18	-0.103673	-0.41694	0.682263	0,07	**93,11**
Serum aCCP level & Annual number of episodes	18	0.294843	1.23424	0.234936	0,23	**77,46**
CRP & Annual duration of all episodes	23	-0.306184	-1.4739	0.155342	0,30	**69,81**
CRP & One episode duration	23	-0.291278	-1.3953	0.177506	0,28	**72,35**
CRP & Annual number of episodes	23	-0.127944	-0.59117	0.560716	0,09	**91,05**
DASCRP & Annual duration of all episodes	13	-0.536452	-2.10824	0.058756	0,50	50,13
DASCRP & One episode duration	13	-0.226934	-0.77282	0.455915	0,12	**88,34**
DASCRP & Annual number of episodes	13	-0.473584	-1.78337	0.102107	0,39	**60,97**
DASESR & Annual duration of all episodes	14	-0.468647	-1.83775	0.090972	0,41	**58,80**
DASESR & One episode duration	14	-0.198911	-0.7031	0.495411	0,11	**89,49**
DASESR & Annual number of episodes	14	-0.498961	-1.99447	0.069325	0,47	53,48
ESR & Annual duration of all episodes	24	-0.092593	-0.43617	0.666963	0,07	**92,89**
ESR & One episode duration	24	0.111458	0.52606	0.60411	0,08	**91,89**
ESR & Annual number of episodes	24	-0.193763	-0.92638	0.364298	0,15	**85,04**
HAQ & Annual duration of all episodes	15	-0.658879	-3.15803	0.007554	0,80	19,68
HAQ & One episode duration	15	-0.401471	-1.58049	0.138011	0,33	**67,15**
HAQ & Annual number of episodes	15	-0.542317	-2.32732	0.036746	0,58	41,94
Morning stiffness, hours & Annual duration of all episodes	21	0.03764	0.16419	0.871318	0,05	**94,77**
Morning stiffness, hours & One episode duration	21	0.016656	0.07261	0.942874	0,05	**95,01**
Morning stiffness, hours & Annual number of episodes	21	0.050319	0.21961	0.828515	0,05	**94,52**
Serum RF level & Annual duration of all episodes	21	0.402373	1.91584	0.070558	0,45	54,53
Serum RF level & One episode duration	21	0.486986	2.43039	0.02516	0,63	36,58
Serum RF level & Annual number of episodes	21	0.067794	0.29619	0.770298	0,06	**94,08**

Number of swollen joints (DAS28 joint count) & Annual duration of all episodes	14	0.211863	0.75096	0.467147	0,11	**88,69**
Number of swollen joints (DAS28 joint count) & One episode duration	14	0.185386	0.65352	0.525744	0,10	**90,26**
Number of swollen joints (DAS28 joint count) & Annual number of episodes	14	-0.010299	-0.03568	0.972125	0,05	**95,08**
Number of swollen joints (68 joint count) & Annual duration of all episodes	13	0.119452	0.39903	0.697506	0,07	**93,32**
Number of swollen joints (68 joint count) & One episode duration	13	-0.173913	-0.58573	0.569879	0,09	**91,24**
Number of swollen joints (68 joint count) & Annual number of episodes	13	-0.005634	-0.01869	0.985427	0,05	**95,10**
Number of tender joints (28 joint count) & Annual duration of all episodes	14	-0.2442	-0.87234	0.400139	0,14	**86,43**
Number of tender joints (28 joint count) & One episode duration	14	-0.136164	-0.47612	0.64254	0,07	**92,54**
Number of tender joints (28 joint count) & Annual number of episodes	14	-0.209826	-0.74341	0.471539	0,11	**88,82**
Number of tender joints (68 joint count) & Annual duration of all episodes	14	-0.588106	-2.51891	0.026963	0,63	36,52
Number of tender joints (68 joint count) & One episode duration	14	-0.46276	-1.80832	0.095666	0,40	**59,80**
Number of tender joints (68 joint count) & Annual number of episodes	14	-0.309248	-1.12649	0.281984	0,19	**80,64**
VAS & Annual duration of all episodes	16	-0.572587	-2.61321	0.020445	0,67	32,65
VAS & One episode duration	16	-0.454167	-1.9074	0.077196	0,44	**55,82**
VAS & Annual number of episodes	16	-0.341327	-1.35873	0.195723	0,26	**73,99**
**Chronic bronchitis exacerbations**						
Serum aCCP level & Annual duration of all episodes	6	-0.115954	-0.23348	0.826848	0,06	**94,42**
Serum aCCP level & One episode duration	6	0.091077	0.18291	0.863763	0,05	**94,64**
Serum aCCP level & Annual number of episodes	6	-0.654654	-1.73205	0.158302	0,31	**68,72**
CRP & Annual duration of all episodes	10	0.400652	1.23682	0.251227	0,22	**78,39**
CRP & One episode duration	10	0.191824	0.55283	0.595488	0,08	**91,73**
CRP & Annual number of episodes	10	0.195508	0.56386	0.588302	0,08	**91,60**
DASCRP & Annual duration of all episodes	5	0.615587	1.35296	0.268998	0,21	**79,03**
DASCRP & One episode duration	5	0.615587	1.35296	0.268998	0,21	**79,03**
DASCRP & Annual number of episodes	5	NA	NA	NA	NA	NA
DASESR & Annual duration of all episodes	6	-0.086966	-0.17459	0.86988	0,05	**94,67**
DASESR & One episode duration	6	-0.086966	-0.17459	0.86988	0,05	**94,67**

DASESR & Annual number of episodes	6	NA	NA	NA	NA	NA
ESR & Annual duration of all episodes	11	0.139588	0.42291	0.682289	0,07	**93,12**
ESR & One episode duration	11	-0.057741	-0.17351	0.866089	0,05	**94,78**
ESR & Annual number of episodes	11	0.129875	0.39295	0.703499	0,07	**93,40**
HAQ & Annual duration of all episodes	5	0.666886	1.55011	0.218894	0,25	**75,07**
HAQ & One episode duration	5	0.631579	1.41095	0.25308	0,22	**77,88**
HAQ & Annual number of episodes	5	0.725476	1.82574	0.16537	0,31	**69,36**
Morning stiffness, hours & Annual duration of all episodes	12	0.12524	0.39919	0.698148	0,07	**93,33**
Morning stiffness, hours & One episode duration	12	0.045128	0.14285	0.889245	0,05	**94,88**
Morning stiffness, hours & Annual number of episodes	12	0.137851	0.44013	0.669207	0,07	**92,94**
Serum RF level & Annual duration of all episodes	11	NA	NA	NA	NA	NA
Serum RF level & One episode duration	11	NA	NA	NA	NA	NA
Serum RF level & Annual number of episodes	11	NA	NA	NA	NA	NA
Number of swollen joints (DAS28 joint count) & Annual duration of all episodes	6	0.602941	1.51154	0.205184	0,26	**74,04**
Number of swollen joints (DAS28 joint count) & One episode duration	6	0.582154	1.43198	0.225416	0,24	**75,90**
Number of swollen joints (DAS28 joint count) & Annual number of episodes	6	0.664211	1.77705	0.1502	0,32	**67,62**
Number of swollen joints (68 joint count) & Annual duration of all episodes	5	0.666886	1.55011	0.218894	0,25	**75,07**
Number of swollen joints (68 joint count) & One episode duration	5	0.631579	1.41095	0.25308	0,22	**77,88**
Number of swollen joints (68 joint count) & Annual number of episodes	5	0.725476	1.82574	0.16537	0,31	**69,36**
Number of tender joints (28 joint count) & Annual duration of all episodes	6	0.764706	2.37346	0.076532	0,47	53,25
Number of tender joints (28 joint count) & One episode duration	6	0.776206	2.46227	0.069521	0,49	51,22
Number of tender joints (28 joint count) & Annual number of episodes	6	0.531369	янв.45	0.277963	0,20	**79,90**
Number of tender joints (68 joint count) & Annual duration of all episodes	6	0.617647	1.57071	0.191342	0,27	**72,63**
Number of tender joints (68 joint count) & One episode duration	6	0.716498	2.05421	0.109167	0,39	**60,84**
Number of tender joints (68 joint count) & Annual number of episodes	6	-0.132842	-0.26806	0.801909	0,06	**94,24**
VAS & Annual duration of all episodes	7	0.666694	2.00015	0.10192	0,40	**59,70**
VAS & One episode duration	7	0.691023	2.13767	0.08557	0,44	**56,02**

VAS & Annual number of episodes	7	0.408248	1	0.363217	0,15	**84,70**
**HSV exacerbation**						
Serum aCCP level & Annual duration of all episodes	54	-0.27296	-2.04604	0.045825	0,52	47,77
Serum aCCP level & One episode duration	54	-0.169014	-1.23657	0.221803	0,23	**76,73**
Serum aCCP level & Annual number of episodes	54	-0.255541	-1.90601	0.062183	0,47	53,14
CRP & Annual duration of all episodes	63	0.065933	0.51608	0.607667	0,08	**91,94**
CRP & One episode duration	63	0.006166	0.04816	0.961748	0,05	**95,00**
CRP & Annual number of episodes	63	0.05869	0.45917	0.647742	0,07	**92,59**
DASCRP & Annual duration of all episodes	30	0.183048	0.98525	0.332941	0,16	**83,63**
DASCRP & One episode duration	30	0.208412	1.12757	0.269071	0,20	**80,04**
DASCRP & Annual number of episodes	30	0.105318	0.56041	0.579659	0,09	**91,43**
DASESR & Annual duration of all episodes	54	-0.042857	-0.30933	0.758304	0,06	**93,93**
DASESR & One episode duration	54	0.122924	0.89319	0.37587	0,14	**85,59**
DASESR & Annual number of episodes	54	-0.127209	-0.92483	0.359327	0,15	**84,90**
ESR &Annual duration of all episodes	69	-0.336034	-2.92038	0.004759	0,82	18,45
ESR & One episode duration	69	-0.19627	-1.6384	0.106026	0,37	**63,09**
ESR &Annual number of episodes	69	-0.366752	-3.22685	0.001938	0,88	11,80
HAQ & Annual duration of all episodes	51	-0.16386	-1.16274	0.250568	0,21	**78,89**
HAQ & One episode duration	51	-0.109393	-0.77037	0.444777	0,12	**88,06**
HAQ & Annual number of episodes	51	-0.128631	-0.90796	0.368344	0,15	**85,28**
Morning stiffness, hours & Annual duration of all episodes	66	-0.056493	-0.45267	0.652317	0,07	**92,66**
Morning stiffness, hours & One episode duration	66	-0.138074	-1.11527	0.268905	0,20	**80,15**
Morning stiffness, hours & Annual number of episodes	66	-0.035067	-0.28071	0.779839	0,06	**94,12**
Serum RF level & Annual duration of all episodes	64	0.056138	0.44273	0.659504	0,07	**92,76**
Serum RF level & One episode duration	64	0.064204	0.50659	0.614242	0,08	**92,05**
Serum RF level & Annual number of episodes	64	0.005408	0.04258	0.966172	0,05	**95,00**
Number of swollen joints (DAS28 joint count) & Annual duration of all episodes	37	0.12129	0.7229	0.474544	0,11	**88,94**

Number of swollen joints (DAS28 joint count) & One episode duration	37	0.098673	0.58662	0.561224	0,09	**91,05**
Number of swollen joints (DAS28 joint count) & Annual number of episodes	37	0.13588	0.8114	0.422619	0,13	**87,32**
Number of swollen joints (68 joint count) & Annual duration of all episodes	37	0.051864	0.30724	0.760479	0,06	**93,96**
Number of swollen joints (68 joint count) & One episode duration	37	0.066779	0.39595	0.694542	0,07	**93,24**
Number of swollen joints (68 joint count) & Annual number of episodes	37	0.035139	0.20802	0.836423	0,05	**94,55**
Number of tender joints (28 joint count) & Annual duration of all episodes	37	-0.056886	-0.33709	0.738064	0,06	**93,74**
Number of tender joints (28 joint count) & One episode duration	37	-0.095881	-0.56987	0.572408	0,09	**91,28**
Number of tender joints (28 joint count) & Annual number of episodes	37	0.029588	0.17512	0.861991	0,05	**94,69**
Number of tender joints (68 joint count) & Annual duration of all episodes	37	-0.307679	-1.91306	0.06395	0,47	53,36
Number of tender joints (68 joint count) & One episode duration	37	-0.335345	-2.10587	0.04246	0,54	46,11
Number of tender joints (68 joint count) & Annual number of episodes	37	-0.190593	-1.14862	0.258504	0,21	**79,38**
VAS & Annual duration of all episodes	62	-0.056468	-0.4381	0.662889	0,07	**92,81**
VAS & One episode duration	62	-0.00262	-0.02029	0.983879	0,05	**95,02**
VAS & Annual number of episodes	62	-0.053573	-0.41557	0.679204	0,07	**93,03**
**Skin and soft tissue infections**						
Serum aCCP level & Annual duration of all episodes	13	0.225968	0.76935	0.457887	0,12	**88,40**
Serum aCCP level & One episode duration	13	-0.474766	-1.78911	0.101135	0,39	**60,78**
Serum aCCP level & Annual number of episodes	13	0.391216	1.40989	0.186216	0,27	**72,88**
CRP & Annual duration of all episodes	15	0.173759	0.63617	0.535703	0,10	**90,49**
CRP & One episode duration	15	-0.020264	-0.07308	0.942858	0,05	**95,03**
CRP & Annual number of episodes	15	0.183004	0.67116	0.513865	0,10	**89,97**
DASCRP &Annual duration of all episodes	7	0.234244	0.53877	0.613155	0,08	**92,00**
DASCRP & One episode duration	7	-0.181848	-0.41352	0.696364	0,07	**93,26**
DASCRP & Annual number of episodes	7	0.316228	0.74536	0.48959	0,11	**89,22**
DASESR & Annual duration of all episodes	12	-0.003559	-0.01126	0.991241	0,05	**95,11**
DASESR & One episode duration	12	-0.218046	-0.70652	0.495995	0,11	**89,49**
DASESR & Annual number of episodes	12	0.070489	0.22346	0.827675	0,05	**94,55**

ESR & Annual duration of all episodes	15	-0.289407	-1.09012	0.295454	0,19	**81,48**
ESR & One episode duration	15	0.341414	1.30968	0.212978	0,24	**75,55**
ESR & Annual number of episodes	15	-0.388946	-1.52222	0.151901	0,31	**69,04**
HAQ & Annual duration of all episodes	9	0.221295	0.60038	0.567177	0,09	**91,16**
HAQ & One episode duration	9	-0.17324	-0.46539	0.655787	0,07	**92,74**
HAQ & Annual number of episodes	9	0.230046	0.62542	0.551533	0,09	**90,83**
Morning stiffness, hours & Annual duration of all episodes	12	0.135474	0.43239	0.674631	0,07	**93,02**
Morning stiffness, hours & One episode duration	12	0.248659	0.81183	0.435789	0,12	**87,68**
Morning stiffness, hours & Annual number of episodes	12	-0.02956	-0.09352	0.92734	0,05	**95,01**
Serum RF level & Annual duration of all episodes	15	NA	NA	NA	NA	NA
Serum RF level & One episode duration	15	NA	NA	NA	NA	NA
Serum RF level & Annual number of episodes	15	NA	NA	NA	NA	NA
Number of swollen joints (DAS28 joint count) & Annual duration of all episodes	7	0.165145	0.37442	0.723449	0,06	**93,59**
Number of swollen joints (DAS28 joint count) & One episode duration	7	0.101852	0.22894	0.827986	0,05	**94,52**
Number of swollen joints (DAS28 joint count) & Annual number of episodes	7	0.080508	0.18061	0.863768	0,05	**94,73**
Number of swollen joints (68 joint count) & Annual duration of all episodes	7	0.072727	0.16305	0.876861	0,05	**94,79**
Number of swollen joints (68 joint count) & One episode duration	7	0.238542	0.54925	0.606461	0,08	**91,88**
Number of swollen joints (68 joint count) & Annual number of episodes	7	-0.079772	-0.17895	0.865005	0,05	**94,74**
Number of tender joints (28 joint count) & Annual duration of all episodes	7	0.272727	0.63387	0.554029	0,09	**90,83**
Number of tender joints (28 joint count) & One episode duration	7	-0.027524	-0.06157	0.953291	0,05	**95,04**
Number of tender joints (28 joint count) & Annual number of episodes	7	0.239317	0.55115	0.605256	0,08	**91,86**
Number of tender joints (68 joint count) & Annual duration of all episodes	7	-0.082572	-0.18527	0.860298	0,05	**94,71**
Number of tender joints (68 joint count) & One episode duration	7	-0.462963	-1.16792	0.295496	0,19	**81,14**
Number of tender joints (68 joint count) & Annual number of episodes	7	0.161015	0.3648	0.730182	0,06	**93,66**
VAS & Annual duration of all episodes	13	0.064161	0.21324	0.835042	0,05	**94,60**
VAS & One episode duration	13	-0.070362	-0.23394	0.819325	0,06	**94,49**
VAS & Annual number of episodes	13	0.078698	0.26182	0.798295	0,06	**94,34**

URIab						
Serum aCCP level & Annual duration of all episodes	27	0.10836	0.54501	0.590578	0,08	**91,64**
Serum aCCP levelSerum aCCP level & One episode duration	27	-0.181319	-0.92188	0.365405	0,15	**85,10**
Serum aCCP level & Annual number of episodes	27	0.262071	1.35781	0.186653	0,27	**73,37**
CRP & Annual duration of all episodes	32	-0.04232	-0.232	0.81811	0,06	**94,43**
CRP & One episode duration	32	-0.237214	-1.33745	0.191129	0,26	**73,88**
CRP & Annual number of episodes	32	0.091575	0.50369	0.618157	0,08	**92,13**
DASCRP & Annual duration of all episodes	18	-0.141374	-0.57123	0.575778	0,09	**91,35**
DASCRP & One episode duration	18	-0.276295	-1.14994	0.267066	0,20	**79,77**
DASCRP & Annual number of episodes	18	-0.049061	-0.19648	0.846707	0,05	**94,65**
DASESR &Annual duration of all episodes	30	-0.051208	-0.27132	0.788133	0,06	**94,21**
DASESR & One episode duration	30	-0.317523	-1.77187	0.0873	0,41	**58,97**
DASESR & Annual number of episodes	30	0.231489	1.25913	0.218381	0,24	**76,31**
ESR & Annual duration of all episodes	34	0.256297	1.49994	0.143433	0,31	**68,51**
ESR & One episode duration	34	0.184099	1.05953	0.29729	0,18	**81,77**
ESR & Annual number of episodes	34	0.036313	0.20555	0.838443	0,05	**94,56**
HAQ & Annual duration of all episodes	24	-0.482188	-2.58161	0.017024	0,69	31,12
HAQ & One episode duration	24	-0.473543	-2.52178	0.019419	0,67	32,99
HAQ & Annual number of episodes	24	-0.064758	-0.30438	0.763698	0,06	**94,01**
Morning stiffness, hours & Annual duration of all episodes	30	-0.076092	-0.40381	0.689419	0,07	**93,18**
Morning stiffness, hours & One episode duration	30	0.049588	0.26272	0.794692	0,06	**94,26**
Morning stiffness, hours & Annual number of episodes	30	-0.181241	-0.97519	0.337819	0,16	**83,87**
Serum RF level & Annual duration of all episodes	34	0.331974	1.99083	0.055096	0,49	50,58
Serum RF level & One episode duration	34	0.163915	0.93996	0.354281	0,15	**84,64**
Serum RF level & Annual number of episodes	34	0.166267	0.95382	0.347323	0,16	**84,33**
Number of swollen joints (DAS28 joint count) & Annual duration of all episodes	21	0.155021	0.68399	0.502239	0,10	**89,68**
Number of swollen joints (DAS28 joint count) & One episode duration	21	0.200067	0.89007	0.384562	0,14	**85,87**

Number of swollen joints (DAS28 joint count) & Annual number of episodes	21	-0.045215	-0.19729	0.845695	0,05	**94,63**
Number of swollen joints (68 joint count) & Annual duration of all episodes	21	0.023394	0.102	0.919826	0,05	**94,95**
Number of swollen joints (68 joint count) & One episode duration	21	0.127153	0.55878	0.582836	0,09	**91,49**
Number of swollen joints (68 joint count) & Annual number of episodes	21	-0.179905	-0.79719	0.435188	0,12	**87,71**
Number of tender joints (28 joint count) & Annual duration of all episodes	21	-0.140079	-0.61667	0.544771	0,09	**90,70**
Number of tender joints (28 joint count) & One episode duration	21	0.065916	0.28795	0.776503	0,06	**94,13**
Number of tender joints (28 joint count) & Annual number of episodes	21	-0.348595	-1.62118	0.121457	0,35	**64,98**
Number of tender joints (68 joint count) & Annual duration of all episodes	21	-0.456465	-2.23625	0.037527	0,57	43,12
Number of tender joints (68 joint count) & One episode duration	21	-0.160756	-0.70995	0.486355	0,11	**89,26**
Number of tender joints (68 joint count) & Annual number of episodes	21	-0.415497	-1.99112	0.061045	0,48	51,82
VAS &Annual duration of all episodes	31	-0.261741	-1.46043	0.154922	0,30	**69,92**
VAS & One episode duration	31	-0.219744	-1.21301	0.234915	0,22	**77,64**
VAS & Annual number of episodes	31	-0.068761	-0.37117	0.713211	0,07	**93,47**
**V-URI**						
Serum aCCP level & Annual duration of all episodes	73	-0.133671	-1.13653	0.259556	0,20	**79,54**
Serum aCCP level & One episode duration	73	-0.05456	-0.46042	0.646622	0,07	**92,57**
Serum aCCP level & Annual number of episodes	73	-0.131363	-1.11656	0.267946	0,20	**80,09**
CRP & Annual duration of all episodes	85	0.106836	0.97893	0.33046	0,16	**83,60**
CRP & One episode duration	86	0.024317	0.22293	0.824129	0,06	**94,45**
CRP & Annual number of episodes	86	0.100336	0.92426	0.357999	0,15	**84,86**
DASCRP & Annual duration of all episodes	50	-0.051712	-0.35875	0.721353	0,06	**93,55**
DASCRP & One episode duration	50	0.023045	0.1597	0.873784	0,05	**94,74**
DASCRP & Annual number of episodes	50	-0.006798	-0.0471	0.962628	0,05	**95,01**
DASESR & Annual duration of all episodes	71	0.076762	0.63952	0.5246	0,10	**90,24**
DASESR & One episode duration	72	0.132633	1.11958	0.266722	0,20	**80,01**
DASESR & Annual number of episodes	72	0.056202	0.47097	0.63913	0,08	**92,46**
ESR & Annual duration of all episodes	91	-0.010044	-0.09476	0.924718	0,05	**94,91**

ESR & One episode duration	92	-0.013467	-0.12777	0.898618	0,05	**94,83**
ESR & Annual number of episodes	92	-0.009179	-0.08709	0.930796	0,05	**94,93**
HAQ & Annual duration of all episodes	63	-0.018096	-0.14136	0.888052	0,05	**94,80**
HAQ & One episode duration	63	0.088771	0.69607	0.489029	0,11	**89,35**
HAQ & Annual number of episodes	63	-0.040264	-0.31472	0.754045	0,06	**93,89**
Morning stiffness, hours & Annual duration of all episodes	83	0.042207	0.38021	0.704788	0,07	**93,35**
Morning stiffness, hours & One episode duration	84	-0.051155	-0.46383	0.643995	0,07	**92,53**
Morning stiffness, hours & Annual number of episodes	84	0.107311	0.97738	0.331254	0,16	**83,63**
Serum RF level & Annual duration of all episodes	83	0.111939	1.01382	0.313686	0,17	**82,75**
Serum RF level & One episode duration	84	0.151426	1.38722	0.169135	0,28	**71,86**
Serum RF level & Annual number of episodes	84	0.034182	0.30971	0.757566	0,06	**93,92**
Number of swollen joints (DAS28 joint count) & Annual duration of all episodes	52	-0.003315	-0.02344	0.98139	0,05	**95,02**
Number of swollen joints (DAS28 joint count) & One episode duration	52	0.004152	0.02936	0.976694	0,05	**95,02**
Number of swollen joints (DAS28 joint count) & Annual number of episodes	52	0.056077	0.39715	0.692949	0,07	**93,21**
Number of swollen joints (68 joint count) & Annual duration of all episodes	51	0.055843	0.39151	0.697119	0,07	**93,27**
Number of swollen joints (68 joint count) & One episode duration	51	0.048364	0.33894	0.736101	0,06	**93,71**
Number of swollen joints (68 joint count) & Annual number of episodes	51	0.107329	0.75567	0.453467	0,12	**88,33**
Number of tender joints (28 joint count) & Annual duration of all episodes	52	-0.026474	-0.18727	0.852209	0,05	**94,63**
Number of tender joints (28 joint count) & One episode duration	52	0.071795	0.50898	0.613003	0,08	**92,03**
Number of tender joints (28 joint count) & Annual number of episodes	52	-0.013356	-0.09445	0.925127	0,05	**94,93**
Number of tender joints (68 joint count) & Annual duration of all episodes	52	-0.138638	-0.98988	0.326999	0,17	**83,39**
Number of tender joints (68 joint count) & One episode duration	52	0.058933	0.41745	0.67814	0,07	**93,02**
Number of tender joints (68 joint count) & Annual number of episodes	52	-0.155322	-1.11178	0.271548	0,20	**80,29**
VAS & Annual duration of all episodes	76	-0.064099	-0.55253	0.582248	0,09	**91,47**
VAS & One episode duration	77	0.049499	0.4292	0.66901	0,07	**92,89**
VAS & Annual number of episodes	77	-0.042746	-0.37053	0.712035	0,07	**93,44**
